# Cerebellum-mediated trainability of eye and head movements for dynamic gazing

**DOI:** 10.1371/journal.pone.0224458

**Published:** 2019-11-04

**Authors:** Akiyoshi Matsugi, Naoki Yoshida, Satoru Nishishita, Yohei Okada, Nobuhiko Mori, Kosuke Oku, Shinya Douchi, Koichi Hosomi, Youichi Saitoh

**Affiliations:** 1 Faculty of Rehabilitation, Shijonawate Gakuen University, Hojo, Daitou City, Osaka, Japan; 2 Department of Research, Institute of Rehabilitation Science, Tokuyukai Medical Corporation, Sakuranocho, Toyonaka City, Osaka, Japan; 3 Department of Rehabilitation, Kansai Rehabilitation Hospital, Sakuranocho, Toyonaka City, Osaka, Japan; 4 Faculty of Health Science, Kio University, Umami-naka, Koryo-cho, Kitakatsuragi-gun, Nara, Japan; 5 Neurorehabilitation Research Center of Kio University, Koryo-cho, Kitakatsuragi-gun, Nara, Japan; 6 Department of Neuromodulation and Neurosurgery, Office for University-Industry Collaboration, Osaka University, Osaka, Japan; 7 Department of Neurosurgery, Osaka University Graduate School of Medicine, Osaka, Japan; 8 Department of Rehabilitation, National Hospital Organization Kyoto Medical Center, Hukakusamukaihatacyo, Husimi-ku, Kyoto City, Kyoto, Japan; Tokyo Medical and Dental University, JAPAN

## Abstract

**Objective:**

To investigate whether gaze stabilization exercises (GSEs) improve eye and head movements and whether low-frequency cerebellar repetitive transcranial magnetic stimulation (rTMS) inhibits GSE trainability.

**Methods:**

25 healthy adults (real rTMS, n = 12; sham rTMS, n = 13) were recruited. Real or sham rTMS was performed for 15 min (1 Hz, 900 stimulations). The center of the butterfly coil was set 1 cm below the inion in the real rTMS. Following stimulation, 10 trials of 1 min of a GSE were conducted at 1 min intervals. In the GSE, the subjects were instructed to stand upright and horizontally rotate their heads according to a beeping sound corresponding to 2 Hz and with a gaze point ahead of them. Electrooculograms were used to estimate the horizontal gaze direction of the right eye, and gyroscopic measurements were performed to estimate the horizontal head angular velocity during the GSE trials. The percentage change from the first trial of motion range of the eye and head was calculated for each measurement. The percent change of the eye/head range ratio was calculated to assess the synchronous changes of the eye and head movements as the exercise increased.

**Results:**

Bayesian two-way analysis of variance showed that cerebellar rTMS affected the eye motion range and eye/head range ratio. A post hoc comparison (Bayesian t-test) showed evidence that the eye motion range and eye/head range ratio were reduced in the fifth, sixth, and seventh trials compared with the first trial sham stimulation condition.

**Conclusions:**

GSEs can modulate eye movements with respect to head movements, and the cerebellum may be associated with eye–head coordination trainability for dynamic gazing during head movements.

## Introduction

Eye and head movements are necessary for accurate visual cognition in daily life [1] because the visual target image on the retina changes with head movements [2]. A low accuracy of detection of visual targets during head movements impacts daily living [3]. This accuracy, known as the dynamic gaze ability, can be improved by gaze stabilization exercises (GSEs) in healthy individuals [4]. One of the possible mechanisms for improving the dynamic gaze ability is the modulation of eye movements with respect to head movements. However, the details of this relationship are unclear.

The vestibuloocular reflex contributes to eye movements during head movements [5]. In young adults, GSEs have been shown to modulate the excitability of vestibular reflexes after only 1 min of GSE [6, 7]. Based on previous reports, it was hypothesized that eye and head movements can be changed in the second and subsequent GSE compared to the first 1 min GSE. However, it is unclear whether GSEs also improve eye and head movements during training in young adults. Therefore, the aim of this study was to investigate improvements in eye and head movements during GSE training.

The cerebellum is involved in the coordination of movements [8, 9], and patients with cerebellar ataxia have difficulty in performing smooth eye and head movements [10]. The cerebellum is also involved in the modulation of the vestibuloocular reflex [11, 12], and it was found that lesions in the cerebellum in monkeys also impaired the modulation of this reflex [13]. The cerebellar dorsal vermis is involved in eye and head movements [12]. Single-pulse transcranial magnetic stimulation (TMS) over the inion, which can affect the cerebellum [14, 15], changes the eye–head coordination [16]. These findings indicate that the medial cerebellum, which includes the oculomotor vermis and nearby areas stimulated by TMS, may be involved in eye movements with respect to head movements. Repetitive TMS (rTMS) can modulate cortical activity beyond the stimulation period, and the possible mechanisms underlying the aftereffects of low-frequency rTMS resemble those of long-term depression [17]. rTMS over the medial cerebellum has been shown to disrupt oculomotor adaptation [18]. Based on these findings, it was hypothesized that low-frequency cerebellar rTMS disrupts the effects achieved by eye–head coordination training such as GSEs. In this study, we investigated whether eye and head movements were modulated by repetitive GSEs in the sham-rTMS condition and whether this modulation was affected by low-frequency rTMS over the medial cerebellum.

## Materials and methods

### Participants

25 healthy adults (mean age: 19.6 ± 0.6 years, 14 males) participated in the study. None of the participants had any history of epilepsy or other neurological diseases. The ethics committee of the Shijonawate Gakuen University approved the experimental procedures (approval code: 29–4), and the study was conducted according to the principles and guidelines of the Declaration of Helsinki [19] with the understanding and written consent of each participant.

### General methodology

Participants were allocated to either the sham-rTMS (n = 13) or real-rTMS (n = 12) groups in a block random method order. Sham- or real-rTMS procedures were conducted before GSE training. The range of motion of the eye and the head in the horizontal plane was measured during the GSE.

### Measurement of the range of motion of the eye and head

**Fig 1:**
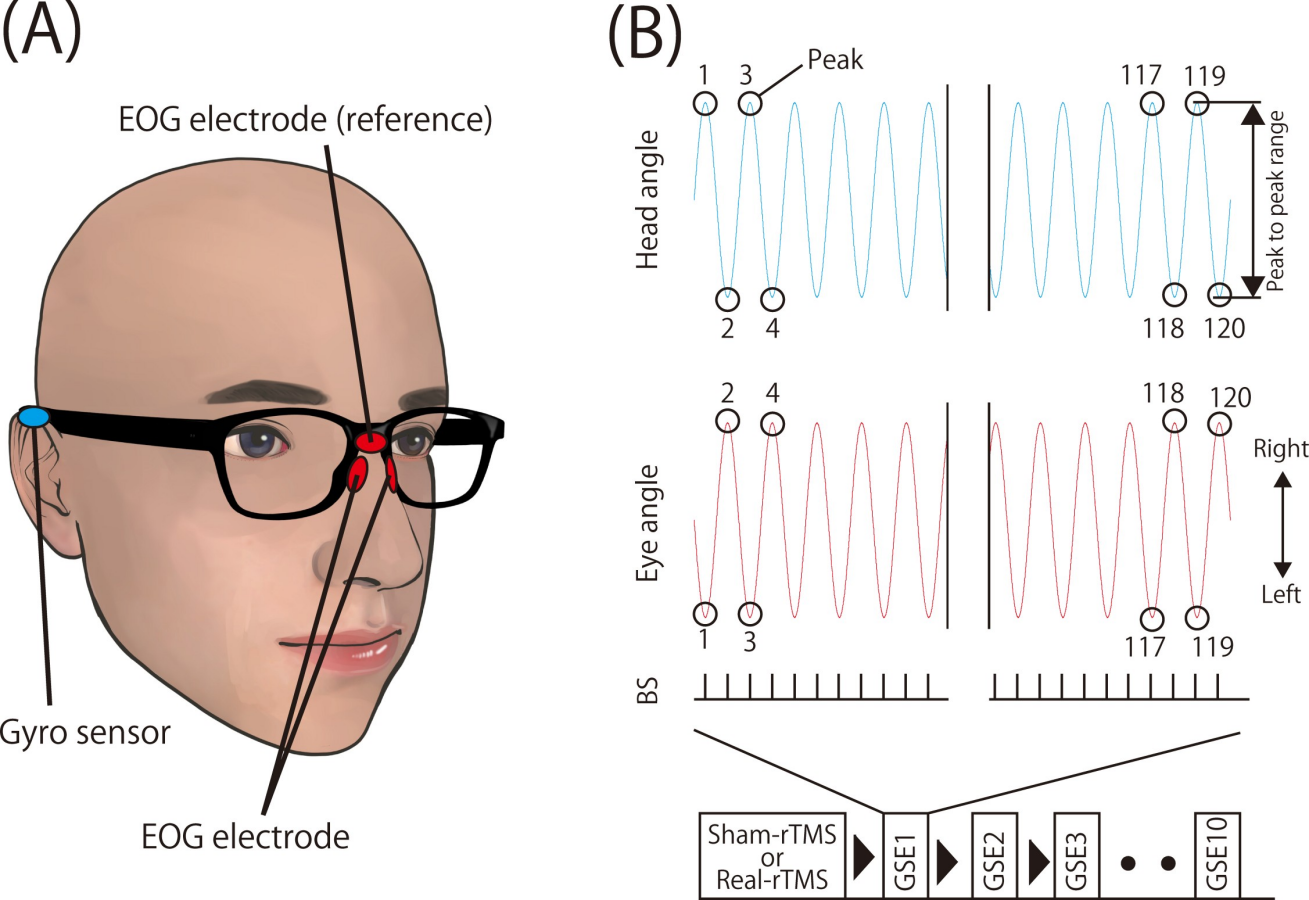
**Experimental setup (A) and data analysis (B).** (A) The black glasses shown are the JINS MEME EOG glasses. The red ovals indicate the EOG electrodes, and the blue ovals indicate the gyroscope sensor attached within the glasses. (B) The blue line indicates the range of the head, and the red line indicates the range of the eye during the GSE. BSs are shown below the range line as short vertical lines. The BS interval was set at 500 ms for 60 s (total number of BSs = 120). The empty circles indicate the peak range, and the number “1” in the figure indicates the first peak of the range of motion. The number continues to the 120th peak. The lower panel in (B) is a schematic of the experimental procedure. The GSE interval was set at 1 min.

In order to estimate the direction of gaze of the right eye in the horizontal plane, electrooculography (EOG) was carried out using JINS MEME EOG glasses (JINS Inc., Tokyo, Japan) [20]. Three dry electrodes were attached to the device and mounted on the nose bridge and nose pads as previously reported [20] (Fig 1A). A high accuracy can be achieved for the EOG data using this dry electrode method and the conventional method of attaching the wet electrode to the outside of the eyes. The former approach was shown to obtain a 6.18% higher accuracy on average compared to the conventional method [20]. In order to record the angle of the head in the horizontal plane, a gyroscope system with wireless JINS MEME glasses [20] was used (Fig 1A). The EOG and gyroscope data were synchronized using the JINS MEME system. The sampling frequency was set at 100 Hz for the EOG and gyroscope sensors. The EOG and gyroscope data were simultaneously transferred from the glasses to a smartphone device using Bluetooth during movement. The data were also transferred to a computer via the ES_R Development Kit application (JINS Inc.). The angle of gaze direction in the horizontal plane was calculated, and 0° was defined as the gaze target in front of the subject when facing the front. A positive degree angle was defined as a deviation to the right side, and a negative degree angle was defined as a deviation to the left side. The angle of the head in the horizontal plane was also calculated, and 0° was defined as the participant facing the target. A positive degree angle was defined as a deviation/rotation of the head to the right. Initially, the data were obtained using a head rotation device (see Supporting Information S1 File) and a plate indicating the angle of 22.5° to the right and left sides (see S2 Fig). The data obtained during the GSE were converted from voltage to angle. In order to estimate the range of motion of the eye and head movements between beeping sounds (BSs), the first range of motion was defined as the range between the first and second peaks, and the second range of motion was defined as the range between the second and third peaks. A single GSE trial involved 120 BSs, and thus a total of 119 points were obtained for the range of motion of the eye and head movements in each GSE trial.

### GSE

Before the GSE, the ability of the subjects to the target at the horizontal head rotation position was confirmed; all subjects could see the target. The subjects were then instructed to stand in an upright position and to repetitively rotate their heads to the right and left in accordance with a 2 Hz BS for 1 min while gazing at a visual target placed 1 m in front of them [4, 6, 7, 21]. The direction of the initial movement was decided by the participants. The subjects were instructed to rotate their heads with the maximum angle that can meet the gaze target [6]. 10 GSE trials were conducted at 1 min intervals (Fig 1B). EOG and gyroscope data were recorded during all tasks.

### Cerebellar rTMS

The participants were asked to lie in a prone position on a bed. A magnetic stimulator (MagPro Compact; MagVenture, Farum, Denmark) was used to deliver TMS to the medial cerebellum using a butterfly coil (MC-B70; MagVenture). It has been reported in previous studies that a butterfly coil, in an eight-shaped figure, used for cerebellar stimulation, resulted in long-lasting inhibitory effects [22, 23]. It has also been shown in previous studies that positioning the center of the coil junction at 1 cm below the inion position leads to the modulation of vestibular and ocular motor functions [16, 18, 24]. The coil junction was, therefore, set at this position to stimulate the central cerebellar areas [14]. These findings indicated that rTMS with a butterfly coil can induce long-lasting inhibition of cerebellar function with respect to oculomotor adaptation. The direction of current in the coil was set downward, in order to deliver an upward current in the brain [25]. It has been shown in previous studies that this direction is effective for cerebellar stimulation [8, 26–30]. The TMS intensity was set at 50% of the maximum stimulator output, similar to that in previous studies investigating cerebellar function and vestibular reflexes [18, 22, 24]. The interstimulus interval was set at 1 s, and 900 pulses were delivered [23, 31]. rTMS can lead to long-lasting aftereffects in the brain [17]. Popa et al. reported that the administration of 1 Hz rTMS (900 pulses) over the cerebellar hemisphere effect on the cerebellar output measured by a paired stimulation method (cerebellar brain inhibition) lasted for 30 min [23]. Jenkinson et al. reported that 1 Hz rTMS (120 pulses) over the inion disrupted oculomotor adaptation, and the aftereffects of rTMS lasted about 10 min [18]. Therefore, 1 min GSE was conducted 10 times immediately after the administration of the conditioning stimulation. The coil was held at a 90° angle from the scalp over the inion while delivering sham TMS [8, 32], which involves the application of auditory stimulation associated with TMS without actual brain stimulation caused by changing magnetic fields.

**Fig 2:**
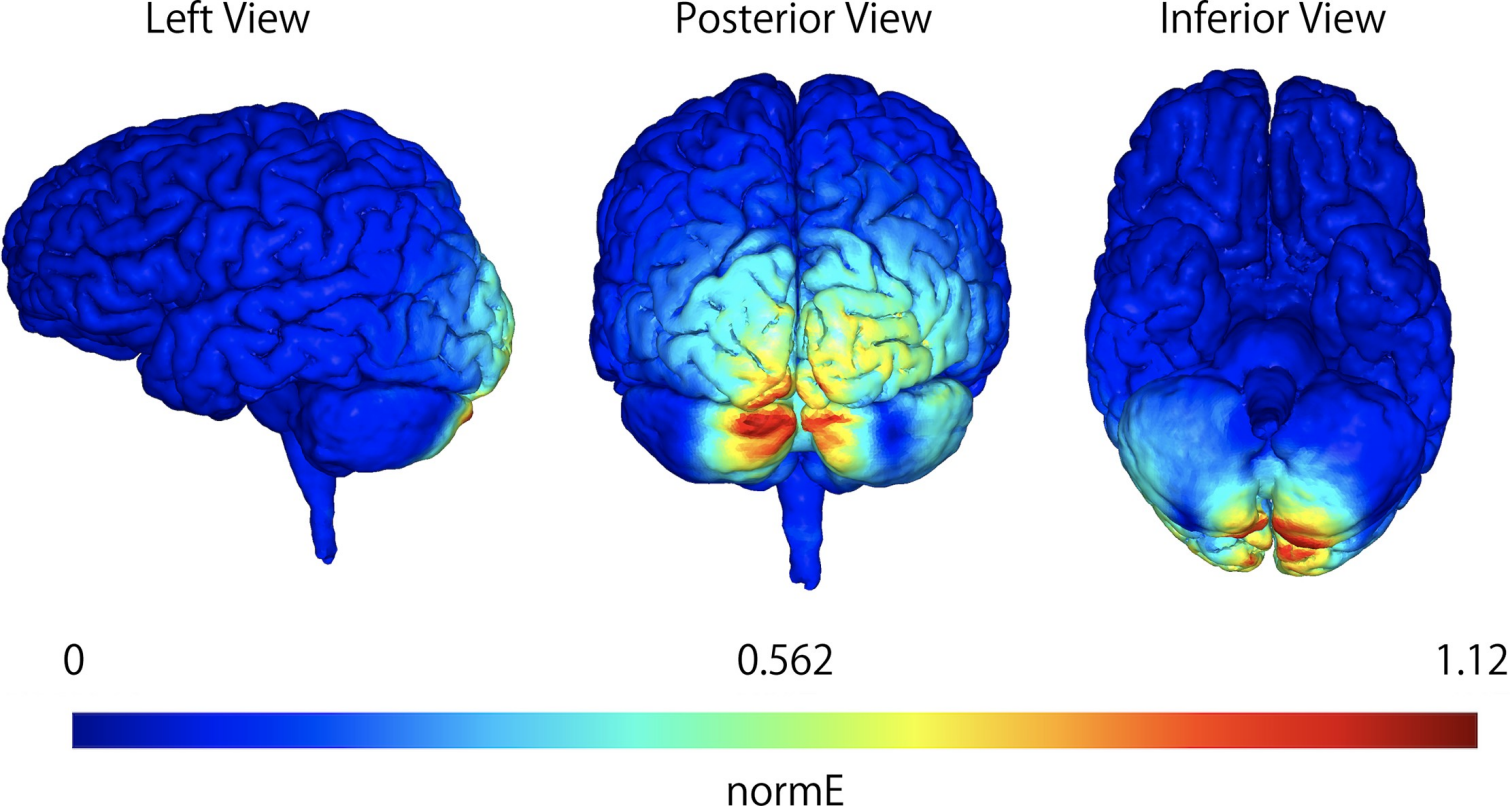
Simulation of the electric field induced by TMS. Electric field induced by TMS using butterfly coils in the coronal, sagittal, and horizontal views. The scale represents normE which is the magnitude of the electric field (V/m) induced by the TMS over the site at 1 cm below the inion. The affected sites are cerebellar structures. TMS: transcranial magnetic stimulation.

Electric field stimulation of the neuronal structures was performed using SimNIBS software (version 2.1.1) [33] with default head models (Fig 2). Biological tissue conductivity values were included in the software version and were set as 0.465 S/m (scalp), 0.01 S/m (bone), 0.5 S/m (eyeballs), 1.654 S/m (cerebrospinal fluid), 0.275 S/m (gray matter), and 0.126 S/m (white matter) [34]. The aforementioned parameters were set for TMS using the butterfly coil.

### Analysis

In order to estimate the degree of eye–head coordination, the eye/head ratio was calculated as the motion range of the eye divided by the motion range of the head for each motion, and the average value obtained in one GSE trial was used as the representative value for an individual. In order to estimate the change of the eye and head motion range and the eye/head ratio after 10 trials were completed, the percentage change from the first trial was calculated. For example, the percentage change of the eye motion range in the third trial was calculated as follows: (eye motion range in the first trial − eye motion range in the third trial)/(eye motion range in the first trial). Calculations were performed using Microsoft Excel for Mac (version 16.16.10; Microsoft Corp., Redmond, WA, USA) and MATLAB software (version R2014b 8.4.0; MathWorks, Natick, MA, USA) in the offline mode.

Levene’s test was conducted as an assumption check to test for the equality of variances of the effect of stimulation and repetition of trials on the percent change from the first trial in the eye and head motion range and eye/head ratio, before two-way analysis of variance (ANOVA) was conducted. If the variances were not equal, a nonparametric analysis (Kruskal–Wallis test) was used, and results with a p-value of <0.05 were considered to be statistically significant. Bayesian two-way ANOVA was conducted because a Bayesian hypothesis test can provide additional information to assist in the interpretation of null results, and this method is used in standalone analyses [35–37]. If there was strong evidence of an alternative hypothesis, a post hoc comparison (Bayesian t-test) [38, 39] was conducted. Posterior odds were corrected for multiple testing by fixing the prior probability that the null hypothesis holds across all comparisons at 0.5 [38]. Statistical analyses were carried out using the JASP software (version 0.9.2; University of Amsterdam, Amsterdam, the Netherlands) [39]. As in a previous study [35], we used the most common prior model as the default in this software.

We computed the predictive performance of two competing hypotheses: the null hypothesis and the alternative hypothesis, that there is an effect [35]. The Bayes factor (BF) [40] allows researchers to quantify evidence in favor of the null hypothesis [35, 41]. If BF_10_ > 10, we believe that there is strong evidence for accepting the alternative hypothesis [35].

## Results

**Fig 3:**
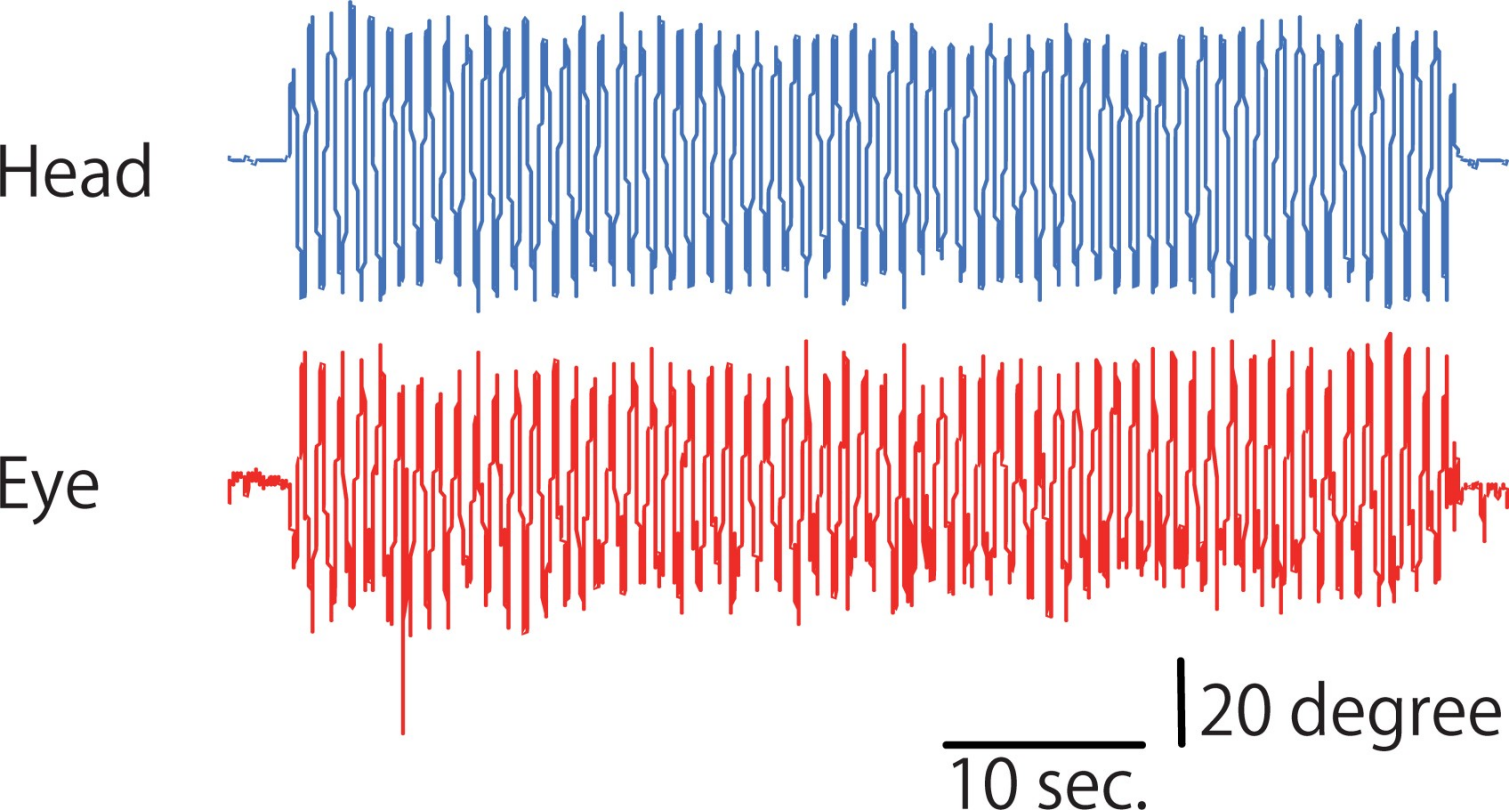
Specimen waveform of the gyroscope and EOG from raw data. The blue and red lines indicate the specimen waveform of the gyroscope (Gyro_Z) and EOG (EOG_H) voltage in the horizontal plane created from raw data extracted from the JINS MEME system during the GSE.

**Fig 4:**
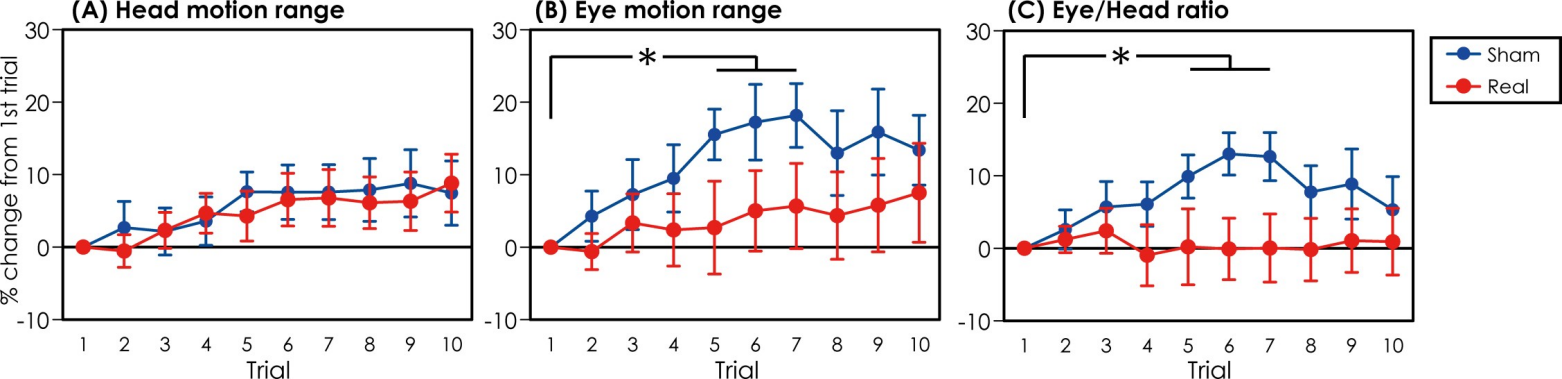
**Motion range of the head (A) and eye (B) and the eye/head ratio (C).** The blank circles (real rTMS) and solid circles (sham rTMS) indicate mean values. The error bars indicate the standard error.

All participants completed all tasks. None of the participants showed any side effects in any of the examinations. Fig 3 shows a typical waveform of the gyroscope and EOG from raw data from the JINS MEME system. All of the raw data in the experiment are attached as Supporting Data. “S” indicates the sham-rTMS group, “R” indicates the real-rTMS group, and the serial number indicates the trial number of the GSE. A summary of the percent changes from the first GSE trial in the range of motion of the eye and head and the eye/head ratio is attached as Supporting Information files. Fig 4 shows the percent change in the range of motion of the eye and head and the eye/head ratio in the sham- and real-rTMS conditions as the mean and standard error. Table 1 shows the results of the tests for equality of variances (Levene’s test). The results indicate that there was no equality of variance between groups for the parametric two-way ANOVA. Therefore, we could not apply parametric two-way ANOVA, and instead nonparametric one-way ANOVA (Kruskal–Wallis test) and Bayesian two-way ANOVA were conducted. Table 2 shows the results of the Kruskal–Wallis test. There was a significant effect of conditioning stimulation observed in the eye motion range and eye/head ratio (p < 0.001), but there was no significant effect on the head motion range (p > 0.05). Table 3 shows the results of the Bayesian two-way ANOVA. The value of BF_10_ was >10 in both the eye motion range and eye/head ratio. Table 4 shows the results of the post hoc comparisons of the percent change in the eye and head movement and eye/head ratio between the sham-rTMS and real-rTMS groups, and BF_10_ > 10 in the eye motion range and eye/head ratio. Table 5 shows the results of the post hoc comparisons of the eye and head motion range and eye/head ratio between trials for each stimulation condition, and BF_10_ > 10 in the comparisons between the first trial and the fifth, sixth, and seventh trials in the sham-rTMS condition for the eye motion range and eye/head ratio.

**Table 1:** Test for equality of variances (Levene’s test) in % change.

	F	df1	df2	p
Head motion range	2.842	19	230	<0.001
Eye motion range	5.435	19	230	<0.001
Eye/head ratio	3.364	19	230	<0.001

**Table 2:** Kruskal–Wallis test in % change.

	Head motion range			Head motion range			Eye/head ratio		
Factor	Statistic	df	p	Statistic	df	p	Statistic	df	p
rTMS	0.425	1	0.515	16.23	1	<0.001	14.97	1	<0.001
Trials	16.605	9	0.055	12.5	9	0.187	6.246	9	0.715

**Table 3:** Bayesian two-way ANOVA (model comparison) in % change.

	Head motion range					Eye motion range					Eye/head ratio				
Models	P(M)	P(M|data)	BF_M_	BF_10_	Error %	P(M)	P(M|data)	BF _M_	BF_10_	Error %	P(M)	P(M|data)	BF _M_	BF _10_	Error %
Null model	0.2	0.7	11	1		0.2	0	0.1	1		0.2	0	0	1	
rTMS	0.2	0.1	0.6	0.2	4.005e -5	0.2	0.9	26	51	1.106e -7	0.2	1	235	390	1.714e -5
Trials	0.2	0.1	0.5	0.2	9.899e -6	0.2	0	0	0.1	2.861e -4	0.2	3.119e -5	1.248e -4	0	6.401e -5
rTMS + Trials	0.2	0	0.1	0	3.9	0.2	0.1	0.5	6.7	1.4	0.2	0	0.1	5.3	1.8
rTMS + Trials + rTMS ✻ Trials	0.2	2.723e -4	0	3.671e -4	0.9	0.2	0	0	0.2	1.7	0.2	7.689e -4	0	0.3	0.9

Note. P(M|data): the probability of the model given the data. BF: Bayesian factor.

**Table 4:** Post hoc comparison between rTMS conditions in % change.

	Prior odds	Posterior odds	BF_10,U_	Error %
Head motion range	1	0.17	0.17	4.005e-5
Eye motion range	1	51.46	51.46	1.106e-7
Eye/head ratio	1	390.5	390.5	1.714e-5

Note. The posterior odds have been corrected for multiple testing by fixing the prior probability that the null hypothesis holds across all comparisons at 0.5 (Westfall, Johnson, and Utts, 1997). Individual comparisons are based on the default t-test with a Cauchy (0, r = 1/sqrt(2)) prior. The “U” in the BF (Bayesian factor) denotes that it is uncorrected.

**Table 5:** Post hoc comparison between trials in % change.

		Head motion range								Eye motion range								Eye/Head ratio							
		Sham				Real				Sham				Real				Sham				Real			
		Prior Odds	Posterior Odds	BF _10, U_	Error %	Prior Odds	Posterior Odds	BF _10, U_	Error %	Prior Odds	Posterior Odds	BF _10, U_	Error %	Prior Odds	Posterior Odds	BF _10, U_	Error %	Prior Odds	Posterior Odds	BF _10, U_	Error %	Prior Odds	Posterior Odds	BF _10, U_	Error %
Trial1	Trial2	0.149	0.066	0.445	2.367e -4	0.149	0.057	0.381	0.016	0.149	0.094	0.634	0.004	0.149	0.057	0.382	0.016	0.149	0.076	0.509	1.653e -4	0.149	0.065	0.439	0.017
	Trial3	0.149	0.063	0.427	2.488e -4	0.149	0.076	0.512	0.019	0.149	0.122	0.819	0.005	0.149	0.071	0.481	0.018	0.149	0.138	0.929	0.003	0.149	0.069	0.464	0.017
	Trial4	0.149	0.082	0.552	0.001	0.149	0.154	1.038	3.970e -4	0.149	0.236	1.588	0.002	0.149	0.06	0.406	0.017	0.149	0.218	1.465	0.002	0.149	0.057	0.38	0.016
	Trial5	0.149	0.752	5.055	5.207e -5	0.149	0.096	0.649	0.005	0.149	19.199	**129.115**	1.252e -4	0.149	0.059	0.398	0.016	0.149	1.977	**13.294**	1.342e -4	0.149	0.056	0.373	0.016
	Trial6	0.149	0.226	1.52	0.002	0.149	0.172	1.157	0.002	0.149	1.921	**12.915**	1.261e -4	0.149	0.075	0.502	0.018	0.149	19.857	**133.538**	6.834e -6	0.149	0.055	0.373	0.016
	Trial7	0.149	0.225	1.51	0.002	0.149	0.16	1.073	8.042e -4	0.149	10.05	**67.584**	5.370e -5	0.149	0.078	0.523	0.019	0.149	5.171	**34.774**	3.443e -5	0.149	0.055	0.373	0.016
	Trial8	0.149	0.177	1.188	2.143e -4	0.149	0.156	1.052	5.519e -4	0.149	0.305	2.051	0.002	0.149	0.067	0.451	0.017	0.149	0.269	1.806	0.002	0.149	0.056	0.373	0.016
	Trial9	0.149	0.194	1.302	9.737e -4	0.149	0.132	0.891	0.001	0.149	0.628	4.222	9.564e -5	0.149	0.074	0.501	0.018	0.149	0.179	1.203	3.102e -4	0.149	0.057	0.381	0.016
	Trial_10	0.149	0.148	0.999	0.001	0.149	0.296	1.99	0.002	0.149	0.737	4.954	0.003	0.149	0.086	0.577	0.002	0.149	0.089	0.597	0.002	0.149	0.056	0.378	0.016
Trial2	Trial3	0.149	0.054	0.364	5.319e -6	0.149	0.072	0.487	0.018	0.149	0.059	0.398	1.321e -4	0.149	0.072	0.482	0.018	0.149	0.065	0.434	2.541e -4	0.149	0.058	0.389	0.016
	Trial4	0.149	0.055	0.367	4.663e -6	0.149	0.12	0.806	0.003	0.149	0.073	0.489	3.811e -6	0.149	0.062	0.415	0.017	0.149	0.071	0.475	7.335e -5	0.149	0.06	0.405	0.017
	Trial5	0.149	0.084	0.562	0.001	0.149	0.091	0.612	0.004	0.149	0.336	2.263	0.001	0.149	0.06	0.406	0.017	0.149	0.175	1.18	1.713e -4	0.149	0.056	0.378	0.016
	Trial6	0.149	0.075	0.501	7.478e -5	0.149	0.146	0.983	1.079e -5	0.149	0.246	1.653	0.002	0.149	0.076	0.509	0.018	0.149	0.566	3.803	1.554e -4	0.149	0.057	0.384	0.016
	Trial7	0.149	0.075	0.502	7.642e -5	0.149	0.141	0.948	3.094e -4	0.149	0.455	3.059	4.541e -4	0.149	0.079	0.53	0.019	0.149	0.368	2.475	0.001	0.149	0.057	0.381	0.016
	Trial8	0.149	0.074	0.497	3.620e -5	0.149	0.134	0.904	0.001	0.149	0.098	0.662	0.004	0.149	0.069	0.461	0.017	0.149	0.087	0.584	0.002	0.149	0.057	0.386	0.016
	Trial9	0.149	0.08	0.539	7.497e -4	0.149	0.122	0.819	0.003	0.149	0.151	1.017	0.001	0.149	0.076	0.51	0.018	0.149	0.086	0.58	0.002	0.149	0.056	0.373	0.016
	Trial_10	0.149	0.07	0.469	1.162e -4	0.149	0.236	1.585	0.003	0.149	0.127	0.852	0.004	0.149	0.087	0.585	0.003	0.149	0.059	0.4	1.426e -4	0.149	0.056	0.374	0.016
Trial3	Trial4	0.149	0.056	0.376	2.326e -5	0.149	0.064	0.433	0.017	0.149	0.056	0.378	3.171e -5	0.149	0.056	0.376	0.016	0.149	0.054	0.364	6.079e -6	0.149	0.065	0.434	0.017
	Trial5	0.149	0.099	0.666	0.004	0.149	0.06	0.404	0.017	0.149	0.109	0.731	0.005	0.149	0.056	0.374	0.016	0.149	0.073	0.493	1.551e -5	0.149	0.058	0.392	0.016
	Trial6	0.149	0.083	0.561	0.001	0.149	0.078	0.521	0.019	0.149	0.11	0.743	0.005	0.149	0.057	0.381	0.016	0.149	0.136	0.914	0.003	0.149	0.06	0.405	0.017
	Trial7	0.149	0.083	0.561	0.001	0.149	0.078	0.523	0.019	0.149	0.147	0.99	0.001	0.149	0.058	0.388	0.016	0.149	0.114	0.768	0.005	0.149	0.059	0.398	0.016
	Trial8	0.149	0.082	0.548	9.779e -4	0.149	0.073	0.493	0.018	0.149	0.067	0.448	2.271e -4	0.149	0.056	0.376	0.016	0.149	0.057	0.386	6.596e -5	0.149	0.061	0.407	0.017
	Trial9	0.149	0.089	0.6	0.003	0.149	0.072	0.484	0.018	0.149	0.086	0.579	0.002	0.149	0.058	0.388	0.016	0.149	0.06	0.403	1.561e -4	0.149	0.057	0.382	0.016
	Trial_10	0.149	0.076	0.511	1.830e -4	0.149	0.11	0.741	0.005	0.149	0.073	0.488	5.114e -6	0.149	0.061	0.413	0.017	0.149	0.054	0.363	6.618e -6	0.149	0.057	0.383	0.016
Trial4	Trial5	0.149	0.075	0.501	7.362e -5	0.149	0.056	0.374	0.016	0.149	0.08	0.54	7.565e -4	0.149	0.056	0.373	0.016	0.149	0.072	0.486	1.214e -5	0.149	0.056	0.377	0.016
	Trial6	0.149	0.068	0.457	1.852e -4	0.149	0.059	0.397	0.016	0.149	0.085	0.57	0.002	0.149	0.058	0.391	0.016	0.149	0.141	0.95	0.002	0.149	0.056	0.376	0.016
	Trial7	0.149	0.068	0.458	1.841e -4	0.149	0.06	0.401	0.017	0.149	0.105	0.709	0.005	0.149	0.059	0.399	0.016	0.149	0.116	0.778	0.005	0.149	0.056	0.377	0.016
	Trial8	0.149	0.068	0.456	1.923e -4	0.149	0.058	0.387	0.016	0.149	0.059	0.394	1.060e -4	0.149	0.057	0.382	0.016	0.149	0.056	0.38	3.794e -5	0.149	0.056	0.376	0.016
	Trial9	0.149	0.073	0.492	1.257e -5	0.149	0.058	0.389	0.016	0.149	0.07	0.474	8.455e -5	0.149	0.059	0.398	0.016	0.149	0.059	0.396	1.180e -4	0.149	0.058	0.389	0.016
	Trial_10	0.149	0.065	0.434	2.541e -4	0.149	0.072	0.487	0.018	0.149	0.061	0.411	1.996e -4	0.149	0.063	0.427	0.017	0.149	0.054	0.365	4.366e -6	0.149	0.057	0.386	0.016
Trial5	Trial6	0.149	0.054	0.363	7.377e -6	0.149	0.06	0.402	0.017	0.149	0.055	0.373	1.487e -5	0.149	0.057	0.384	0.016	0.149	0.066	0.446	2.355e -4	0.149	0.056	0.373	0.016
	Trial7	0.149	0.054	0.363	7.344e -6	0.149	0.06	0.406	0.017	0.149	0.059	0.394	1.065e -4	0.149	0.058	0.39	0.016	0.149	0.062	0.417	2.246e -4	0.149	0.055	0.373	0.016
	Trial8	0.149	0.054	0.363	6.925e -6	0.149	0.058	0.392	0.016	0.149	0.057	0.382	4.849e -5	0.149	0.056	0.378	0.016	0.149	0.058	0.392	9.745e -5	0.149	0.056	0.374	0.016
	Trial9	0.149	0.055	0.369	6.987e -6	0.149	0.059	0.394	0.016	0.149	0.054	0.363	6.917e -6	0.149	0.058	0.389	0.016	0.149	0.055	0.367	4.718e -6	0.149	0.056	0.375	0.016
	Trial_10	0.149	0.054	0.363	7.175e -6	0.149	0.073	0.488	0.018	0.149	0.057	0.381	4.242e -5	0.149	0.061	0.411	0.017	0.149	0.07	0.471	1.007e -4	0.149	0.056	0.375	0.016
Trial6	Trial7	0.149	0.054	0.363	7.403e -6	0.149	0.056	0.373	0.016	0.149	0.054	0.365	4.464e -6	0.149	0.056	0.374	0.016	0.149	0.054	0.364	6.157e -6	0.149	0.055	0.373	0.016
	Trial8	0.149	0.054	0.363	6.836e -6	0.149	0.056	0.374	0.016	0.149	0.06	0.405	1.676e -4	0.149	0.056	0.374	0.016	0.149	0.086	0.579	0.002	0.149	0.055	0.373	0.016
	Trial9	0.149	0.055	0.368	6.048e -6	0.149	0.056	0.373	0.016	0.149	0.055	0.367	4.262e -6	0.149	0.056	0.374	0.016	0.149	0.066	0.443	2.423e -4	0.149	0.056	0.378	0.016
	Trial_10	0.149	0.054	0.363	7.346e -6	0.149	0.059	0.398	0.016	0.149	0.06	0.405	1.671e -4	0.149	0.057	0.384	0.016	0.149	0.112	0.755	0.005	0.149	0.056	0.377	0.016
Trial7	Trial8	0.149	0.054	0.363	6.877e -6	0.149	0.056	0.375	0.016	0.149	0.065	0.437	2.524e -4	0.149	0.056	0.377	0.016	0.149	0.078	0.522	3.667e -4	0.149	0.056	0.373	0.016
	Trial9	0.149	0.055	0.368	5.850e -6	0.149	0.056	0.374	0.016	0.149	0.056	0.376	2.430e -5	0.149	0.055	0.373	0.016	0.149	0.063	0.423	2.423e -4	0.149	0.056	0.377	0.016
	Trial_10	0.149	0.054	0.363	7.307e -6	0.149	0.058	0.392	0.016	0.149	0.066	0.443	2.431e -4	0.149	0.056	0.379	0.016	0.149	0.1	0.671	0.005	0.149	0.056	0.376	0.016
Trial8	Trial9	0.149	0.054	0.365	4.305e -6	0.149	0.056	0.373	0.016	0.149	0.056	0.38	3.720e -5	0.149	0.056	0.377	0.016	0.149	0.055	0.367	4.689e -6	0.149	0.056	0.379	0.016
	Trial_10	0.149	0.054	0.363	6.493e -6	0.149	0.061	0.41	0.017	0.149	0.054	0.363	6.880e -6	0.149	0.058	0.39	0.016	0.149	0.058	0.387	7.178e -5	0.149	0.056	0.377	0.016
Trial9	Trial_10	0.149	0.055	0.369	6.402e -6	0.149	0.06	0.4	0.017	0.149	0.056	0.377	2.954e -5	0.149	0.056	0.378	0.016	0.149	0.06	0.403	1.587e -4	0.149	0.056	0.373	0.016

Note. The posterior odds have been corrected for multiple testing by fixing the prior probability that the null hypothesis holds across all comparisons as 0.5 (Westfall, Johnson, and Utts, 1997). Individual comparisons are based on the default t-test with a Cauchy (0, r = 1/sqrt(2)) prior. The “U” in the BF (Bayesian factor) denotes that it is uncorrected.

## Discussion

The aim of this study was to investigate whether the GSE improves eye and head movements and whether low-frequency cerebellar rTMS inhibits this GSE trainability. Our results indicate that there was strong evidence that the percent change from the first trial in the range of motion of the eye and the eye/head ratio was reduced by the GSE in the sham-rTMS conditions, but this was not the case for the range of motion of the head. There was no evidence for an effect of GSEs on the head or eye motion range or eye/head ratio in the real-rTMS conditions. There was strong evidence for a reduction of the eye motion range and eye/head ratio in the fifth, sixth, and seventh trials compared with the first trial only in the sham-rTMS condition. These findings indicate that the GSE reduced the range of eye motion with respect to head movements and low-frequency rTMS over the medial cerebellum disrupted the modulation caused by training.

The visual target image on the retina deviates with head movements; therefore, anticipatory and reflexive eye movements are necessary for accurate visual cognition [1]. Accuracy in the dynamic gaze during head movements is improved by GSEs in healthy subjects [4], an observation that suggests that the dynamic gaze can be trained to detect visual targets during movements more accurately. In the present study, we asked our subjects to conduct maximal rotation of their heads in order to see the target and found that the range of eye motion during head movements was reduced by repetitive GSEs under the sham-rTMS conditions. Therefore, we speculate that a reduction in the range of eye motion may increase the accuracy of the dynamic gaze; the eye motion in the first trial might have frequently overshot the target.

The range of eye motion was changed without changing the range of head motion. Therefore, the reduction of the eye/head ratio depends upon the reduction of the eye motion range, because the head motion range does not change. One reason for this may be that the participants were trying to follow the instructions regarding head motion range.

Modulation of the eye motion range during head movements was not observed after low-frequency cerebellar rTMS. The cerebellum contributes to adaptive changes in the vestibuloocular reflex, as shown by the observation that cerebellar lesions disturb long-term adaptive changes in the vestibular reflex [11, 13]. Further, the vermal cerebellum contributes to saccadic adaptation [42]. Low-frequency cerebellar rTMS reduces cerebellar brain inhibition immediately after stimulation [23], indicating that it inhibits the excitability of the cerebellar cortex or deep nuclei. Low-frequency rTMS has been shown to disturb eye movements [18] and motor adaptation [25]. However, our result of reduced eye motion range in the sham rTMS condition indicates the overshot target, suggesting that cerebellar rTMS does not disturb eye movement immediately. Therefore, the modulation of eye movements with respect to head movements by GSEs for dynamic gaze may be associated with cerebellar function.

This study has some limitations. The sample size was small, which may account for the inequality of variances. We conducted a Bayesian analysis [35] as a complementary analysis technique. However, in order to confirm the reproducibility of the results, it will be necessary to perform additional experiments in the future, with a larger sample size. There are also some methodological considerations. In order to reduce the effect of bias in the individual abilities of eye and head movements, the participants were randomly allocated to stimulation groups. However, there may have been some bias due to the differences in the ability of the eye and head movements between individuals. We did not measure the performance of the eye and head movements during GSEs before the conditioning stimulation, because we considered that the aftereffects of GSEs before stimulation might have remained and affected the trainability of the eye and head movements. We did not measure the accuracy of detection of visual targets during rapid head rotation tasks, as was done in previous studies [4], and therefore could not estimate the change of the ability of dynamic gaze itself. We can only speculate that the dynamic gaze ability is increased as seen in the previous study [4], based on the result of the change of eye movements.

Our findings (that the range of motion of the eye is modulated with respect to head movements for dynamic gaze) may contribute to the development of a standardized methodology for vestibular rehabilitation. Patients with reduced cerebellar function may show poor response to GSEs during dynamic gazing. Further studies are needed to investigate the effects of GSEs on eye and head movements in patients with cerebellar dysfunction in clinical practice.

In conclusion, in this study, we found that training to increase the accuracy of dynamic gaze modulates eye movements with respect to head movements. However, these effects were not present after low-frequency cerebellar rTMS. This finding indicates that the cerebellum contributes to the trainability of eye movements for dynamic gaze.

## Supporting information

S1 Fig Head rotation device. (TIF)

S2 Fig Plate for gaze and head angle. (TIF)

S1 File Statistical analysis: Test for the equality of variances (Levene’s test), Kruskal–Wallis test, Bayesian two-way ANOVA (model comparison), and post hoc comparison (between rTMS conditions) in head motion range. (JASP)

S2 File Statistical analysis: Test for the equality of variances (Levene’s test), Kruskal–Wallis test, Bayesian two-way ANOVA (model comparison), and post hoc comparison (between rTMS conditions) in eye motion range. (JASP)

S3 File Statistical analysis: Test for the equality of variances (Levene’s test), Kruskal–Wallis test, Bayesian two-way ANOVA (model comparison), and post hoc comparisons between rTMS conditions in eye/head ratio. (JASP)

S4 File Statistical analysis: Post hoc comparisons between trials in head motion range of sham rTMS. (JASP)

S5 File Statistical analysis: Post hoc comparisons between trials in head motion range of real rTMS. (JASP)

S6 File Statistical analysis: Post hoc comparisons between trials in eye motion range of sham rTMS. (JASP)

S7 File Statistical analysis: Post hoc comparisons between trials in eye motion range of real rTMS. (JASP)

S8 File Statistical analysis: Post hoc comparisons between trials in eye/head ratio of sham rTMS. (JASP)

S9 File Statistical analysis: Post hoc comparisons between trials in eye/head ratio of real rTMS. (JASP)
