# Supplementary figures and images for: Cerebellum-mediated trainability of eye and head movements for dynamic gazing
Source: PLoS One. 2019 Nov 4;14(11):e0224458. doi: 10.1371/journal.pone.0224458 (PMC6827899; doi:10.1371/journal.pone.0224458)

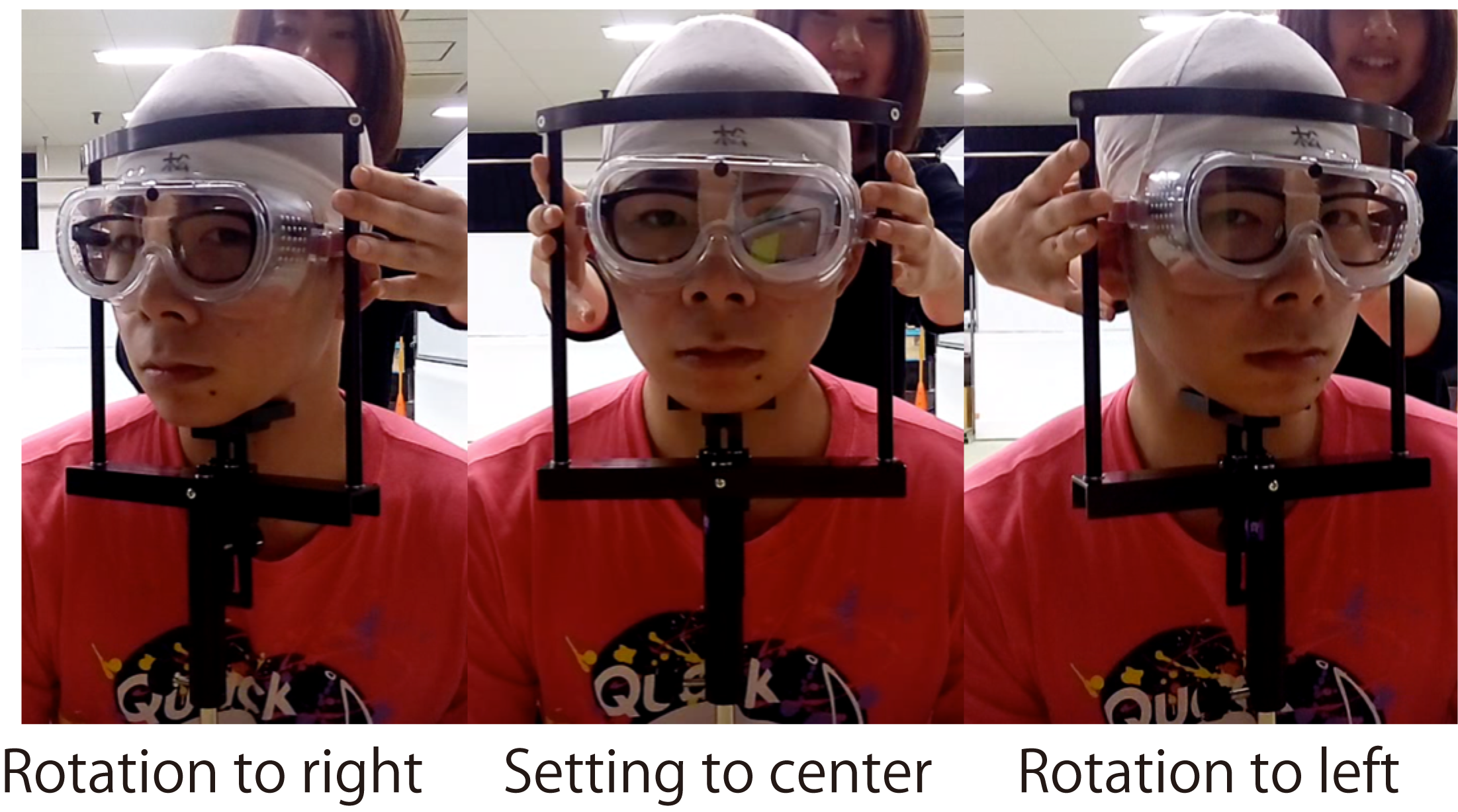

Supplement: S1 Fig — (TIF) [file pone.0224458.s001.tif]

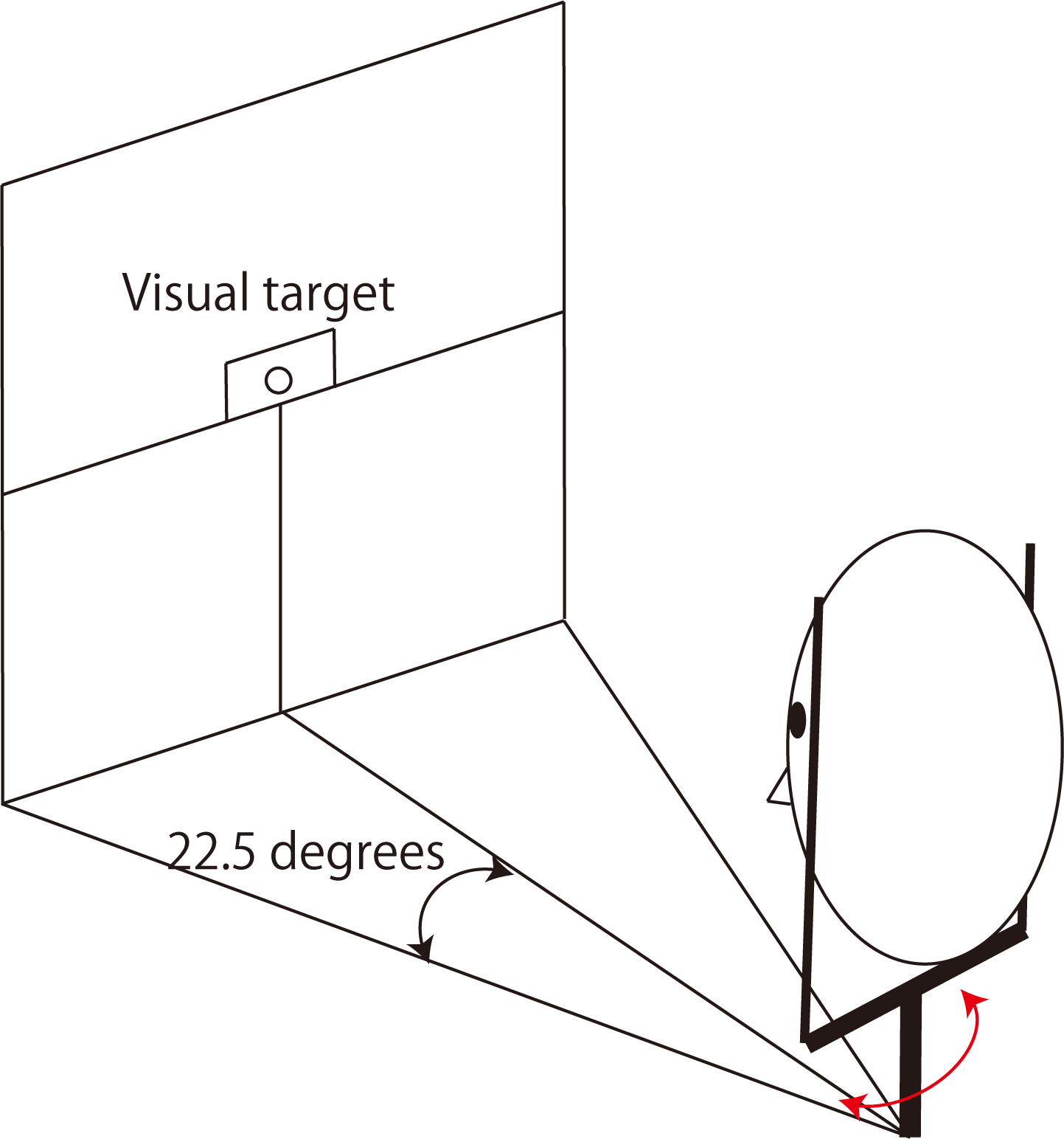

Supplement: S2 Fig — (TIF) [file pone.0224458.s002.tif]

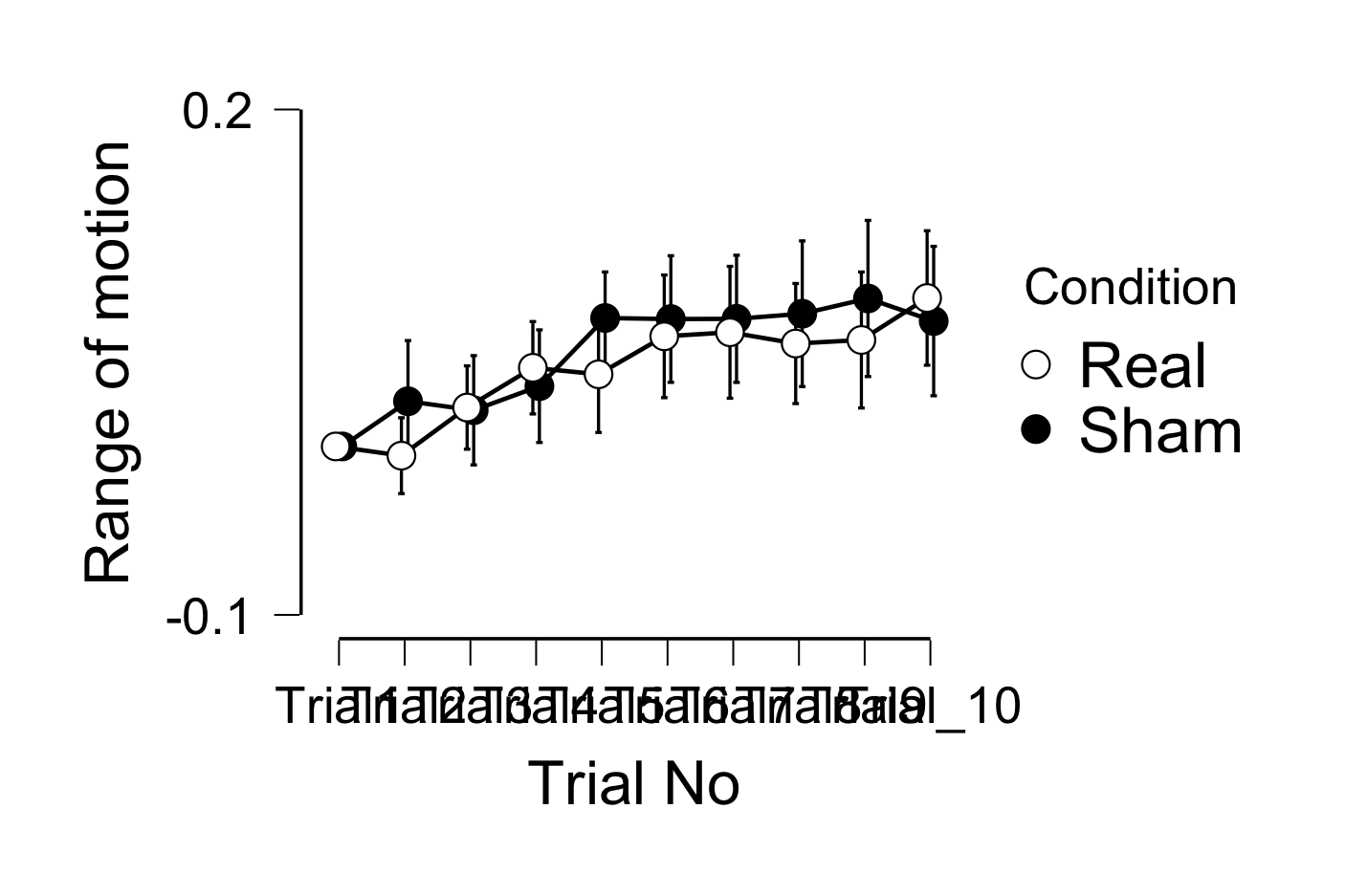

Supplement: S1 File — (JASP) [file pone.0224458.s003.jasp › resources/0/_3.png]

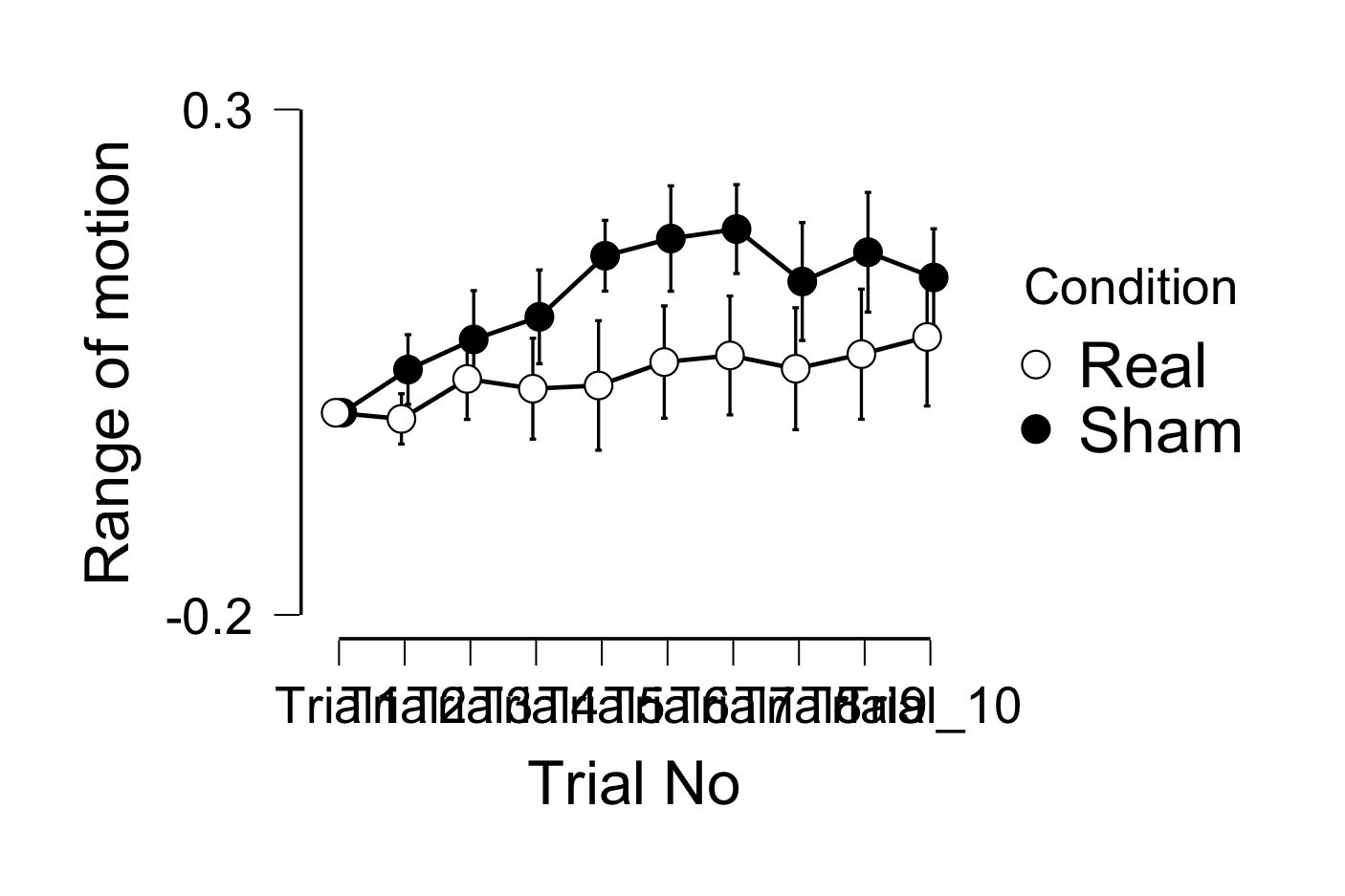

Supplement: S2 File — (JASP) [file pone.0224458.s004.jasp › resources/3/_0.png]

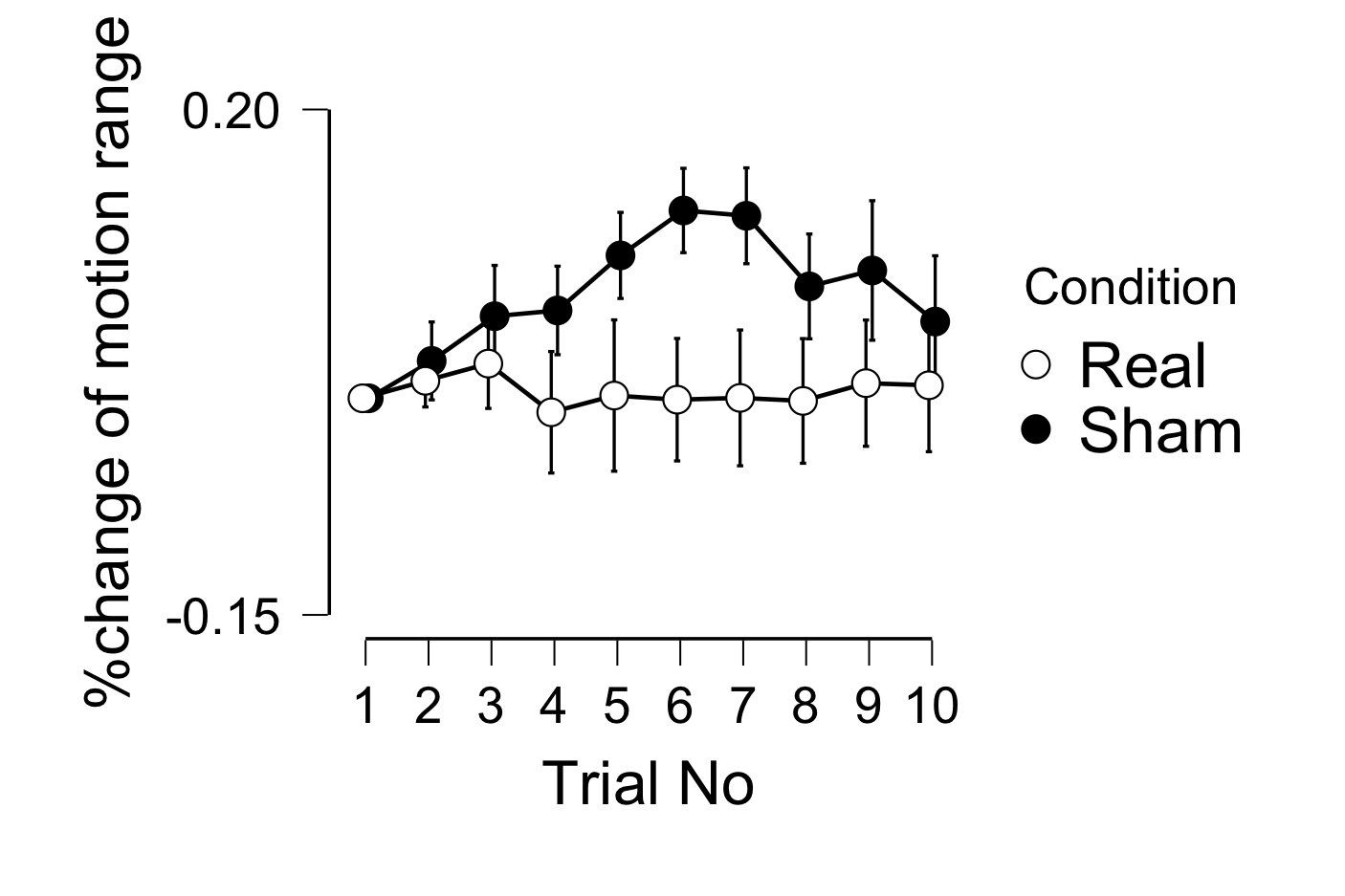

Supplement: S3 File — (JASP) [file pone.0224458.s005.jasp › resources/0/_3.png]
