# Supplementary material for: Cerebellum-mediated trainability of eye and head movements for dynamic gazing
Source: PLoS One. 2019 Nov 4;14(11):e0224458. doi: 10.1371/journal.pone.0224458 (PMC6827899; doi:10.1371/journal.pone.0224458)
Supplement: S1 File — (JASP) [file pone.0224458.s003.jasp › index.html]

JASP 


# Results

## ANOVA

| ANOVA - Range of motion | | | | | | | | | | | |
| --- | --- | --- | --- | --- | --- | --- | --- | --- | --- | --- | --- |
| Cases | | Sum of Squares | | df | | Mean Square | | F | | p | |
| Condition |  | 0.006 |  | 1 |  | 0.006 |  | 0.421 |  | 0.517 |  |
| Trial No |  | 0.197 |  | 9 |  | 0.022 |  | 1.478 |  | 0.157 |  |
| Condition ✻ Trial No |  | 0.016 |  | 9 |  | 0.002 |  | 0.120 |  | 0.999 |  |
| Residual |  | 3.409 |  | 230 |  | 0.015 |  |  |  |  |  |
|  | | | | | | | | | | | |
|  |  |  |  |  |  |  |  |  |  |  |  |
| --- | --- | --- | --- | --- | --- | --- | --- | --- | --- | --- | --- |
| *Note.*  Type III Sum of Squares | | | | | | | | | | | |

### Assumption Checks

| Test for Equality of Variances (Levene's) | | | | | | | |
| --- | --- | --- | --- | --- | --- | --- | --- |
| F | | df1 | | df2 | | p | |
| 2.842 |  | 19 |  | 230 |  | < .001 |  |
|  | | | | | | | |

| Kruskal-Wallis Test | | | | | | | |
| --- | --- | --- | --- | --- | --- | --- | --- |
| Factor | | Statistic | | df | | p | |
| Condition |  | 0.425 |  | 1 |  | 0.515 |  |
| Trial No |  | 16.605 |  | 9 |  | 0.055 |  |
|  | | | | | | | |

### Descriptives

#### Descriptives Plot

## Bayesian ANOVA

| Model Comparison - Range of motion | | | | | | | | | | | |
| --- | --- | --- | --- | --- | --- | --- | --- | --- | --- | --- | --- |
| Models | | P(M) | | P(M|data) | | BF M | | BF 10 | | error % | |
| Null model |  | 0.200 |  | 0.743 |  | 11.543 |  | 1.000 |  |  |  |
| Condition |  | 0.200 |  | 0.126 |  | 0.577 |  | 0.170 |  | 4.005e -5 |  |
| Trial No |  | 0.200 |  | 0.112 |  | 0.506 |  | 0.151 |  | 9.899e -6 |  |
| Condition + Trial No |  | 0.200 |  | 0.019 |  | 0.076 |  | 0.025 |  | 1.002 |  |
| Condition + Trial No + Condition  ✻  Trial No |  | 0.200 |  | 2.801e -4 |  | 0.001 |  | 3.772e -4 |  | 1.741 |  |
|  | | | | | | | | | | | |

### Post Hoc Tests

| Post Hoc Comparisons - Condition | | | | | | | | | | | |
| --- | --- | --- | --- | --- | --- | --- | --- | --- | --- | --- | --- |
|  | |  | | Prior Odds | | Posterior Odds | | BF 10, U | | error % | |
| Real |  | Sham |  | 1.000 |  | 0.170 |  | 0.170 |  | 4.005e -5 |  |
|  | | | | | | | | | | | |
|  |  |  |  |  |  |  |  |  |  |  |  |
| --- | --- | --- | --- | --- | --- | --- | --- | --- | --- | --- | --- |
| *Note.*  The posterior odds have been corrected for multiple testing by fixing to 0.5 the prior probability that the null hypothesis holds across all comparisons (Westfall, Johnson, & Utts, 1997). Individual comparisons are based on the default t-test with a Cauchy (0, r = 1/sqrt(2)) prior. The "U" in the Bayes factor denotes that it is uncorrected. | | | | | | | | | | | |
