# Supplementary material for: Cerebellum-mediated trainability of eye and head movements for dynamic gazing
Source: PLoS One. 2019 Nov 4;14(11):e0224458. doi: 10.1371/journal.pone.0224458 (PMC6827899; doi:10.1371/journal.pone.0224458)
Supplement: S3 File — (JASP) [file pone.0224458.s005.jasp › index.html]

JASP 


# Results

## ANOVA

| ANOVA - %change of motion range | | | | | | | | | | | |
| --- | --- | --- | --- | --- | --- | --- | --- | --- | --- | --- | --- |
| Cases | | Sum of Squares | | df | | Mean Square | | F | | p | |
| Condition |  | 0.282 |  | 1 |  | 0.282 |  | 16.812 |  | < .001 |  |
| Trial No |  | 0.091 |  | 9 |  | 0.010 |  | 0.602 |  | 0.795 |  |
| Condition ✻ Trial No |  | 0.111 |  | 9 |  | 0.012 |  | 0.734 |  | 0.677 |  |
| Residual |  | 3.864 |  | 230 |  | 0.017 |  |  |  |  |  |
|  | | | | | | | | | | | |
|  |  |  |  |  |  |  |  |  |  |  |  |
| --- | --- | --- | --- | --- | --- | --- | --- | --- | --- | --- | --- |
| *Note.*  Type III Sum of Squares | | | | | | | | | | | |

### Assumption Checks

| Test for Equality of Variances (Levene's) | | | | | | | |
| --- | --- | --- | --- | --- | --- | --- | --- |
| F | | df1 | | df2 | | p | |
| 3.364 |  | 19 |  | 230 |  | < .001 |  |
|  | | | | | | | |

| Kruskal-Wallis Test | | | | | | | |
| --- | --- | --- | --- | --- | --- | --- | --- |
| Factor | | Statistic | | df | | p | |
| Condition |  | 14.970 |  | 1 |  | < .001 |  |
| Trial No |  | 6.246 |  | 9 |  | 0.715 |  |
|  | | | | | | | |

### Descriptives

#### Descriptives Plot

## Bayesian ANOVA

| Model Comparison - %change of motion range | | | | | | | | | | | |
| --- | --- | --- | --- | --- | --- | --- | --- | --- | --- | --- | --- |
| Models | | P(M) | | P(M|data) | | BF M | | BF 10 | | error % | |
| Null model |  | 0.200 |  | 0.003 |  | 0.010 |  | 1.000 |  |  |  |
| Condition |  | 0.200 |  | 0.983 |  | 234.650 |  | 390.498 |  | 1.714e -5 |  |
| Trial No |  | 0.200 |  | 3.119e -5 |  | 1.248e -4 |  | 0.012 |  | 6.401e -5 |  |
| Condition + Trial No |  | 0.200 |  | 0.013 |  | 0.055 |  | 5.339 |  | 1.775 |  |
| Condition + Trial No + Condition  ✻  Trial No |  | 0.200 |  | 7.689e -4 |  | 0.003 |  | 0.305 |  | 0.936 |  |
|  | | | | | | | | | | | |

### Post Hoc Tests

| Post Hoc Comparisons - Condition | | | | | | | | | | | |
| --- | --- | --- | --- | --- | --- | --- | --- | --- | --- | --- | --- |
|  | |  | | Prior Odds | | Posterior Odds | | BF 10, U | | error % | |
| Real |  | Sham |  | 1.000 |  | 390.5 |  | 390.5 |  | 1.714e -5 |  |
|  | | | | | | | | | | | |
|  |  |  |  |  |  |  |  |  |  |  |  |
| --- | --- | --- | --- | --- | --- | --- | --- | --- | --- | --- | --- |
| *Note.*  The posterior odds have been corrected for multiple testing by fixing to 0.5 the prior probability that the null hypothesis holds across all comparisons (Westfall, Johnson, & Utts, 1997). Individual comparisons are based on the default t-test with a Cauchy (0, r = 1/sqrt(2)) prior. The "U" in the Bayes factor denotes that it is uncorrected. | | | | | | | | | | | |
