# Supplementary material for: Cerebellum-mediated trainability of eye and head movements for dynamic gazing
Source: PLoS One. 2019 Nov 4;14(11):e0224458. doi: 10.1371/journal.pone.0224458 (PMC6827899; doi:10.1371/journal.pone.0224458)
Supplement: S4 File — (JASP) [file pone.0224458.s006.jasp › index.html]

JASP 


# Results

## Bayesian ANOVA

| Model Comparison - Range of motion | | | | | | | | | | | |
| --- | --- | --- | --- | --- | --- | --- | --- | --- | --- | --- | --- |
| Models | | P(M) | | P(M|data) | | BF M | | BF 10 | | error % | |
| Null model |  | 0.500 |  | 0.951 |  | 19.295 |  | 1.000 |  |  |  |
| Trial No |  | 0.500 |  | 0.049 |  | 0.052 |  | 0.052 |  | 0.003 |  |
|  | | | | | | | | | | | |

### Post Hoc Tests

| Post Hoc Comparisons - Trial No | | | | | | | | | | | |
| --- | --- | --- | --- | --- | --- | --- | --- | --- | --- | --- | --- |
|  | |  | | Prior Odds | | Posterior Odds | | BF 10, U | | error % | |
| Trial1 |  | Trial2 |  | 0.149 |  | 0.066 |  | 0.445 |  | 2.367e -4 |  |
|  |  | Trial3 |  | 0.149 |  | 0.063 |  | 0.427 |  | 2.488e -4 |  |
|  |  | Trial4 |  | 0.149 |  | 0.082 |  | 0.552 |  | 0.001 |  |
|  |  | Trial5 |  | 0.149 |  | 0.752 |  | 5.055 |  | 5.207e -5 |  |
|  |  | Trial6 |  | 0.149 |  | 0.226 |  | 1.520 |  | 0.002 |  |
|  |  | Trial7 |  | 0.149 |  | 0.225 |  | 1.510 |  | 0.002 |  |
|  |  | Trial8 |  | 0.149 |  | 0.177 |  | 1.188 |  | 2.143e -4 |  |
|  |  | Trial9 |  | 0.149 |  | 0.194 |  | 1.302 |  | 9.737e -4 |  |
|  |  | Trial\_10 |  | 0.149 |  | 0.148 |  | 0.999 |  | 0.001 |  |
| Trial2 |  | Trial3 |  | 0.149 |  | 0.054 |  | 0.364 |  | 5.319e -6 |  |
|  |  | Trial4 |  | 0.149 |  | 0.055 |  | 0.367 |  | 4.663e -6 |  |
|  |  | Trial5 |  | 0.149 |  | 0.084 |  | 0.562 |  | 0.001 |  |
|  |  | Trial6 |  | 0.149 |  | 0.075 |  | 0.501 |  | 7.478e -5 |  |
|  |  | Trial7 |  | 0.149 |  | 0.075 |  | 0.502 |  | 7.642e -5 |  |
|  |  | Trial8 |  | 0.149 |  | 0.074 |  | 0.497 |  | 3.620e -5 |  |
|  |  | Trial9 |  | 0.149 |  | 0.080 |  | 0.539 |  | 7.497e -4 |  |
|  |  | Trial\_10 |  | 0.149 |  | 0.070 |  | 0.469 |  | 1.162e -4 |  |
| Trial3 |  | Trial4 |  | 0.149 |  | 0.056 |  | 0.376 |  | 2.326e -5 |  |
|  |  | Trial5 |  | 0.149 |  | 0.099 |  | 0.666 |  | 0.004 |  |
|  |  | Trial6 |  | 0.149 |  | 0.083 |  | 0.561 |  | 0.001 |  |
|  |  | Trial7 |  | 0.149 |  | 0.083 |  | 0.561 |  | 0.001 |  |
|  |  | Trial8 |  | 0.149 |  | 0.082 |  | 0.548 |  | 9.779e -4 |  |
|  |  | Trial9 |  | 0.149 |  | 0.089 |  | 0.600 |  | 0.003 |  |
|  |  | Trial\_10 |  | 0.149 |  | 0.076 |  | 0.511 |  | 1.830e -4 |  |
| Trial4 |  | Trial5 |  | 0.149 |  | 0.075 |  | 0.501 |  | 7.362e -5 |  |
|  |  | Trial6 |  | 0.149 |  | 0.068 |  | 0.457 |  | 1.852e -4 |  |
|  |  | Trial7 |  | 0.149 |  | 0.068 |  | 0.458 |  | 1.841e -4 |  |
|  |  | Trial8 |  | 0.149 |  | 0.068 |  | 0.456 |  | 1.923e -4 |  |
|  |  | Trial9 |  | 0.149 |  | 0.073 |  | 0.492 |  | 1.257e -5 |  |
|  |  | Trial\_10 |  | 0.149 |  | 0.065 |  | 0.434 |  | 2.541e -4 |  |
| Trial5 |  | Trial6 |  | 0.149 |  | 0.054 |  | 0.363 |  | 7.377e -6 |  |
|  |  | Trial7 |  | 0.149 |  | 0.054 |  | 0.363 |  | 7.344e -6 |  |
|  |  | Trial8 |  | 0.149 |  | 0.054 |  | 0.363 |  | 6.925e -6 |  |
|  |  | Trial9 |  | 0.149 |  | 0.055 |  | 0.369 |  | 6.987e -6 |  |
|  |  | Trial\_10 |  | 0.149 |  | 0.054 |  | 0.363 |  | 7.175e -6 |  |
| Trial6 |  | Trial7 |  | 0.149 |  | 0.054 |  | 0.363 |  | 7.403e -6 |  |
|  |  | Trial8 |  | 0.149 |  | 0.054 |  | 0.363 |  | 6.836e -6 |  |
|  |  | Trial9 |  | 0.149 |  | 0.055 |  | 0.368 |  | 6.048e -6 |  |
|  |  | Trial\_10 |  | 0.149 |  | 0.054 |  | 0.363 |  | 7.346e -6 |  |
| Trial7 |  | Trial8 |  | 0.149 |  | 0.054 |  | 0.363 |  | 6.877e -6 |  |
|  |  | Trial9 |  | 0.149 |  | 0.055 |  | 0.368 |  | 5.850e -6 |  |
|  |  | Trial\_10 |  | 0.149 |  | 0.054 |  | 0.363 |  | 7.307e -6 |  |
| Trial8 |  | Trial9 |  | 0.149 |  | 0.054 |  | 0.365 |  | 4.305e -6 |  |
|  |  | Trial\_10 |  | 0.149 |  | 0.054 |  | 0.363 |  | 6.493e -6 |  |
| Trial9 |  | Trial\_10 |  | 0.149 |  | 0.055 |  | 0.369 |  | 6.402e -6 |  |
|  | | | | | | | | | | | |
|  |  |  |  |  |  |  |  |  |  |  |  |
| --- | --- | --- | --- | --- | --- | --- | --- | --- | --- | --- | --- |
| *Note.*  The posterior odds have been corrected for multiple testing by fixing to 0.5 the prior probability that the null hypothesis holds across all comparisons (Westfall, Johnson, & Utts, 1997). Individual comparisons are based on the default t-test with a Cauchy (0, r = 1/sqrt(2)) prior. The "U" in the Bayes factor denotes that it is uncorrected. | | | | | | | | | | | |
