# Supplementary material for: Cerebellum-mediated trainability of eye and head movements for dynamic gazing
Source: PLoS One. 2019 Nov 4;14(11):e0224458. doi: 10.1371/journal.pone.0224458 (PMC6827899; doi:10.1371/journal.pone.0224458)
Supplement: S5 File — (JASP) [file pone.0224458.s007.jasp › index.html]

JASP 


# Results

## Bayesian ANOVA

| Model Comparison - Range of motion | | | | | | | | | | | |
| --- | --- | --- | --- | --- | --- | --- | --- | --- | --- | --- | --- |
| Models | | P(M) | | P(M|data) | | BF M | | BF 10 | | error % | |
| Null model |  | 0.500 |  | 0.923 |  | 11.980 |  | 1.000 |  |  |  |
| Trial No |  | 0.500 |  | 0.077 |  | 0.083 |  | 0.083 |  | 0.002 |  |
|  | | | | | | | | | | | |

### Post Hoc Tests

| Post Hoc Comparisons - Trial No | | | | | | | | | | | |
| --- | --- | --- | --- | --- | --- | --- | --- | --- | --- | --- | --- |
|  | |  | | Prior Odds | | Posterior Odds | | BF 10, U | | error % | |
| Trial1 |  | Trial2 |  | 0.149 |  | 0.057 |  | 0.381 |  | 0.016 |  |
|  |  | Trial3 |  | 0.149 |  | 0.076 |  | 0.512 |  | 0.019 |  |
|  |  | Trial4 |  | 0.149 |  | 0.154 |  | 1.038 |  | 3.970e -4 |  |
|  |  | Trial5 |  | 0.149 |  | 0.096 |  | 0.649 |  | 0.005 |  |
|  |  | Trial6 |  | 0.149 |  | 0.172 |  | 1.157 |  | 0.002 |  |
|  |  | Trial7 |  | 0.149 |  | 0.160 |  | 1.073 |  | 8.042e -4 |  |
|  |  | Trial8 |  | 0.149 |  | 0.156 |  | 1.052 |  | 5.519e -4 |  |
|  |  | Trial9 |  | 0.149 |  | 0.132 |  | 0.891 |  | 0.001 |  |
|  |  | Trial\_10 |  | 0.149 |  | 0.296 |  | 1.990 |  | 0.002 |  |
| Trial2 |  | Trial3 |  | 0.149 |  | 0.072 |  | 0.487 |  | 0.018 |  |
|  |  | Trial4 |  | 0.149 |  | 0.120 |  | 0.806 |  | 0.003 |  |
|  |  | Trial5 |  | 0.149 |  | 0.091 |  | 0.612 |  | 0.004 |  |
|  |  | Trial6 |  | 0.149 |  | 0.146 |  | 0.983 |  | 1.079e -5 |  |
|  |  | Trial7 |  | 0.149 |  | 0.141 |  | 0.948 |  | 3.094e -4 |  |
|  |  | Trial8 |  | 0.149 |  | 0.134 |  | 0.904 |  | 0.001 |  |
|  |  | Trial9 |  | 0.149 |  | 0.122 |  | 0.819 |  | 0.003 |  |
|  |  | Trial\_10 |  | 0.149 |  | 0.236 |  | 1.585 |  | 0.003 |  |
| Trial3 |  | Trial4 |  | 0.149 |  | 0.064 |  | 0.433 |  | 0.017 |  |
|  |  | Trial5 |  | 0.149 |  | 0.060 |  | 0.404 |  | 0.017 |  |
|  |  | Trial6 |  | 0.149 |  | 0.078 |  | 0.521 |  | 0.019 |  |
|  |  | Trial7 |  | 0.149 |  | 0.078 |  | 0.523 |  | 0.019 |  |
|  |  | Trial8 |  | 0.149 |  | 0.073 |  | 0.493 |  | 0.018 |  |
|  |  | Trial9 |  | 0.149 |  | 0.072 |  | 0.484 |  | 0.018 |  |
|  |  | Trial\_10 |  | 0.149 |  | 0.110 |  | 0.741 |  | 0.005 |  |
| Trial4 |  | Trial5 |  | 0.149 |  | 0.056 |  | 0.374 |  | 0.016 |  |
|  |  | Trial6 |  | 0.149 |  | 0.059 |  | 0.397 |  | 0.016 |  |
|  |  | Trial7 |  | 0.149 |  | 0.060 |  | 0.401 |  | 0.017 |  |
|  |  | Trial8 |  | 0.149 |  | 0.058 |  | 0.387 |  | 0.016 |  |
|  |  | Trial9 |  | 0.149 |  | 0.058 |  | 0.389 |  | 0.016 |  |
|  |  | Trial\_10 |  | 0.149 |  | 0.072 |  | 0.487 |  | 0.018 |  |
| Trial5 |  | Trial6 |  | 0.149 |  | 0.060 |  | 0.402 |  | 0.017 |  |
|  |  | Trial7 |  | 0.149 |  | 0.060 |  | 0.406 |  | 0.017 |  |
|  |  | Trial8 |  | 0.149 |  | 0.058 |  | 0.392 |  | 0.016 |  |
|  |  | Trial9 |  | 0.149 |  | 0.059 |  | 0.394 |  | 0.016 |  |
|  |  | Trial\_10 |  | 0.149 |  | 0.073 |  | 0.488 |  | 0.018 |  |
| Trial6 |  | Trial7 |  | 0.149 |  | 0.056 |  | 0.373 |  | 0.016 |  |
|  |  | Trial8 |  | 0.149 |  | 0.056 |  | 0.374 |  | 0.016 |  |
|  |  | Trial9 |  | 0.149 |  | 0.056 |  | 0.373 |  | 0.016 |  |
|  |  | Trial\_10 |  | 0.149 |  | 0.059 |  | 0.398 |  | 0.016 |  |
| Trial7 |  | Trial8 |  | 0.149 |  | 0.056 |  | 0.375 |  | 0.016 |  |
|  |  | Trial9 |  | 0.149 |  | 0.056 |  | 0.374 |  | 0.016 |  |
|  |  | Trial\_10 |  | 0.149 |  | 0.058 |  | 0.392 |  | 0.016 |  |
| Trial8 |  | Trial9 |  | 0.149 |  | 0.056 |  | 0.373 |  | 0.016 |  |
|  |  | Trial\_10 |  | 0.149 |  | 0.061 |  | 0.410 |  | 0.017 |  |
| Trial9 |  | Trial\_10 |  | 0.149 |  | 0.060 |  | 0.400 |  | 0.017 |  |
|  | | | | | | | | | | | |
|  |  |  |  |  |  |  |  |  |  |  |  |
| --- | --- | --- | --- | --- | --- | --- | --- | --- | --- | --- | --- |
| *Note.*  The posterior odds have been corrected for multiple testing by fixing to 0.5 the prior probability that the null hypothesis holds across all comparisons (Westfall, Johnson, & Utts, 1997). Individual comparisons are based on the default t-test with a Cauchy (0, r = 1/sqrt(2)) prior. The "U" in the Bayes factor denotes that it is uncorrected. | | | | | | | | | | | |

## Bayesian Paired Samples T-Test

| Bayesian Paired Samples T-Test | | | | | | | | | |
| --- | --- | --- | --- | --- | --- | --- | --- | --- | --- |
|  | |  | |  | | BF₁₀ | | error % | |
| ... |  | - |  | ... |  |  |  |  |  |
|  | | | | | | | | | |
