# Supplementary material for: Cerebellum-mediated trainability of eye and head movements for dynamic gazing
Source: PLoS One. 2019 Nov 4;14(11):e0224458. doi: 10.1371/journal.pone.0224458 (PMC6827899; doi:10.1371/journal.pone.0224458)
Supplement: S6 File — (JASP) [file pone.0224458.s008.jasp › index.html]

JASP 


# Results

## Bayesian ANOVA

| Model Comparison - Range of motion | | | | | | | | | | | |
| --- | --- | --- | --- | --- | --- | --- | --- | --- | --- | --- | --- |
| Models | | P(M) | | P(M|data) | | BF M | | BF 10 | | error % | |
| Null model |  | 0.500 |  | 0.658 |  | 1.924 |  | 1.000 |  |  |  |
| Trial No |  | 0.500 |  | 0.342 |  | 0.520 |  | 0.520 |  | 1.108e -5 |  |
|  | | | | | | | | | | | |

### Post Hoc Tests

| Post Hoc Comparisons - Trial No | | | | | | | | | | | |
| --- | --- | --- | --- | --- | --- | --- | --- | --- | --- | --- | --- |
|  | |  | | Prior Odds | | Posterior Odds | | BF 10, U | | error % | |
| Trial1 |  | Trial2 |  | 0.149 |  | 0.094 |  | 0.634 |  | 0.004 |  |
|  |  | Trial3 |  | 0.149 |  | 0.122 |  | 0.819 |  | 0.005 |  |
|  |  | Trial4 |  | 0.149 |  | 0.236 |  | 1.588 |  | 0.002 |  |
|  |  | Trial5 |  | 0.149 |  | 19.199 |  | 129.115 |  | 1.252e -4 |  |
|  |  | Trial6 |  | 0.149 |  | 1.921 |  | 12.915 |  | 1.261e -4 |  |
|  |  | Trial7 |  | 0.149 |  | 10.050 |  | 67.584 |  | 5.370e -5 |  |
|  |  | Trial8 |  | 0.149 |  | 0.305 |  | 2.051 |  | 0.002 |  |
|  |  | Trial9 |  | 0.149 |  | 0.628 |  | 4.222 |  | 9.564e -5 |  |
|  |  | Trial\_10 |  | 0.149 |  | 0.737 |  | 4.954 |  | 0.003 |  |
| Trial2 |  | Trial3 |  | 0.149 |  | 0.059 |  | 0.398 |  | 1.321e -4 |  |
|  |  | Trial4 |  | 0.149 |  | 0.073 |  | 0.489 |  | 3.811e -6 |  |
|  |  | Trial5 |  | 0.149 |  | 0.336 |  | 2.263 |  | 0.001 |  |
|  |  | Trial6 |  | 0.149 |  | 0.246 |  | 1.653 |  | 0.002 |  |
|  |  | Trial7 |  | 0.149 |  | 0.455 |  | 3.059 |  | 4.541e -4 |  |
|  |  | Trial8 |  | 0.149 |  | 0.098 |  | 0.662 |  | 0.004 |  |
|  |  | Trial9 |  | 0.149 |  | 0.151 |  | 1.017 |  | 0.001 |  |
|  |  | Trial\_10 |  | 0.149 |  | 0.127 |  | 0.852 |  | 0.004 |  |
| Trial3 |  | Trial4 |  | 0.149 |  | 0.056 |  | 0.378 |  | 3.171e -5 |  |
|  |  | Trial5 |  | 0.149 |  | 0.109 |  | 0.731 |  | 0.005 |  |
|  |  | Trial6 |  | 0.149 |  | 0.110 |  | 0.743 |  | 0.005 |  |
|  |  | Trial7 |  | 0.149 |  | 0.147 |  | 0.990 |  | 0.001 |  |
|  |  | Trial8 |  | 0.149 |  | 0.067 |  | 0.448 |  | 2.271e -4 |  |
|  |  | Trial9 |  | 0.149 |  | 0.086 |  | 0.579 |  | 0.002 |  |
|  |  | Trial\_10 |  | 0.149 |  | 0.073 |  | 0.488 |  | 5.114e -6 |  |
| Trial4 |  | Trial5 |  | 0.149 |  | 0.080 |  | 0.540 |  | 7.565e -4 |  |
|  |  | Trial6 |  | 0.149 |  | 0.085 |  | 0.570 |  | 0.002 |  |
|  |  | Trial7 |  | 0.149 |  | 0.105 |  | 0.709 |  | 0.005 |  |
|  |  | Trial8 |  | 0.149 |  | 0.059 |  | 0.394 |  | 1.060e -4 |  |
|  |  | Trial9 |  | 0.149 |  | 0.070 |  | 0.474 |  | 8.455e -5 |  |
|  |  | Trial\_10 |  | 0.149 |  | 0.061 |  | 0.411 |  | 1.996e -4 |  |
| Trial5 |  | Trial6 |  | 0.149 |  | 0.055 |  | 0.373 |  | 1.487e -5 |  |
|  |  | Trial7 |  | 0.149 |  | 0.059 |  | 0.394 |  | 1.065e -4 |  |
|  |  | Trial8 |  | 0.149 |  | 0.057 |  | 0.382 |  | 4.849e -5 |  |
|  |  | Trial9 |  | 0.149 |  | 0.054 |  | 0.363 |  | 6.917e -6 |  |
|  |  | Trial\_10 |  | 0.149 |  | 0.057 |  | 0.381 |  | 4.242e -5 |  |
| Trial6 |  | Trial7 |  | 0.149 |  | 0.054 |  | 0.365 |  | 4.464e -6 |  |
|  |  | Trial8 |  | 0.149 |  | 0.060 |  | 0.405 |  | 1.676e -4 |  |
|  |  | Trial9 |  | 0.149 |  | 0.055 |  | 0.367 |  | 4.262e -6 |  |
|  |  | Trial\_10 |  | 0.149 |  | 0.060 |  | 0.405 |  | 1.671e -4 |  |
| Trial7 |  | Trial8 |  | 0.149 |  | 0.065 |  | 0.437 |  | 2.524e -4 |  |
|  |  | Trial9 |  | 0.149 |  | 0.056 |  | 0.376 |  | 2.430e -5 |  |
|  |  | Trial\_10 |  | 0.149 |  | 0.066 |  | 0.443 |  | 2.431e -4 |  |
| Trial8 |  | Trial9 |  | 0.149 |  | 0.056 |  | 0.380 |  | 3.720e -5 |  |
|  |  | Trial\_10 |  | 0.149 |  | 0.054 |  | 0.363 |  | 6.880e -6 |  |
| Trial9 |  | Trial\_10 |  | 0.149 |  | 0.056 |  | 0.377 |  | 2.954e -5 |  |
|  | | | | | | | | | | | |
|  |  |  |  |  |  |  |  |  |  |  |  |
| --- | --- | --- | --- | --- | --- | --- | --- | --- | --- | --- | --- |
| *Note.*  The posterior odds have been corrected for multiple testing by fixing to 0.5 the prior probability that the null hypothesis holds across all comparisons (Westfall, Johnson, & Utts, 1997). Individual comparisons are based on the default t-test with a Cauchy (0, r = 1/sqrt(2)) prior. The "U" in the Bayes factor denotes that it is uncorrected. | | | | | | | | | | | |
