# Supplementary material for: Cerebellum-mediated trainability of eye and head movements for dynamic gazing
Source: PLoS One. 2019 Nov 4;14(11):e0224458. doi: 10.1371/journal.pone.0224458 (PMC6827899; doi:10.1371/journal.pone.0224458)
Supplement: S7 File — (JASP) [file pone.0224458.s009.jasp › index.html]

JASP 


# Results

## Bayesian ANOVA

| Model Comparison - Range of motion | | | | | | | | | | | |
| --- | --- | --- | --- | --- | --- | --- | --- | --- | --- | --- | --- |
| Models | | P(M) | | P(M|data) | | BF M | | BF 10 | | error % | |
| Null model |  | 0.500 |  | 0.980 |  | 49.437 |  | 1.000 |  |  |  |
| Trial No |  | 0.500 |  | 0.020 |  | 0.020 |  | 0.020 |  | 3.451e -5 |  |
|  | | | | | | | | | | | |

### Post Hoc Tests

| Post Hoc Comparisons - Trial No | | | | | | | | | | | |
| --- | --- | --- | --- | --- | --- | --- | --- | --- | --- | --- | --- |
|  | |  | | Prior Odds | | Posterior Odds | | BF 10, U | | error % | |
| Trial1 |  | Trial2 |  | 0.149 |  | 0.057 |  | 0.382 |  | 0.016 |  |
|  |  | Trial3 |  | 0.149 |  | 0.071 |  | 0.481 |  | 0.018 |  |
|  |  | Trial4 |  | 0.149 |  | 0.060 |  | 0.406 |  | 0.017 |  |
|  |  | Trial5 |  | 0.149 |  | 0.059 |  | 0.398 |  | 0.016 |  |
|  |  | Trial6 |  | 0.149 |  | 0.075 |  | 0.502 |  | 0.018 |  |
|  |  | Trial7 |  | 0.149 |  | 0.078 |  | 0.523 |  | 0.019 |  |
|  |  | Trial8 |  | 0.149 |  | 0.067 |  | 0.451 |  | 0.017 |  |
|  |  | Trial9 |  | 0.149 |  | 0.074 |  | 0.501 |  | 0.018 |  |
|  |  | Trial\_10 |  | 0.149 |  | 0.086 |  | 0.577 |  | 0.002 |  |
| Trial2 |  | Trial3 |  | 0.149 |  | 0.072 |  | 0.482 |  | 0.018 |  |
|  |  | Trial4 |  | 0.149 |  | 0.062 |  | 0.415 |  | 0.017 |  |
|  |  | Trial5 |  | 0.149 |  | 0.060 |  | 0.406 |  | 0.017 |  |
|  |  | Trial6 |  | 0.149 |  | 0.076 |  | 0.509 |  | 0.018 |  |
|  |  | Trial7 |  | 0.149 |  | 0.079 |  | 0.530 |  | 0.019 |  |
|  |  | Trial8 |  | 0.149 |  | 0.069 |  | 0.461 |  | 0.017 |  |
|  |  | Trial9 |  | 0.149 |  | 0.076 |  | 0.510 |  | 0.018 |  |
|  |  | Trial\_10 |  | 0.149 |  | 0.087 |  | 0.585 |  | 0.003 |  |
| Trial3 |  | Trial4 |  | 0.149 |  | 0.056 |  | 0.376 |  | 0.016 |  |
|  |  | Trial5 |  | 0.149 |  | 0.056 |  | 0.374 |  | 0.016 |  |
|  |  | Trial6 |  | 0.149 |  | 0.057 |  | 0.381 |  | 0.016 |  |
|  |  | Trial7 |  | 0.149 |  | 0.058 |  | 0.388 |  | 0.016 |  |
|  |  | Trial8 |  | 0.149 |  | 0.056 |  | 0.376 |  | 0.016 |  |
|  |  | Trial9 |  | 0.149 |  | 0.058 |  | 0.388 |  | 0.016 |  |
|  |  | Trial\_10 |  | 0.149 |  | 0.061 |  | 0.413 |  | 0.017 |  |
| Trial4 |  | Trial5 |  | 0.149 |  | 0.056 |  | 0.373 |  | 0.016 |  |
|  |  | Trial6 |  | 0.149 |  | 0.058 |  | 0.391 |  | 0.016 |  |
|  |  | Trial7 |  | 0.149 |  | 0.059 |  | 0.399 |  | 0.016 |  |
|  |  | Trial8 |  | 0.149 |  | 0.057 |  | 0.382 |  | 0.016 |  |
|  |  | Trial9 |  | 0.149 |  | 0.059 |  | 0.398 |  | 0.016 |  |
|  |  | Trial\_10 |  | 0.149 |  | 0.063 |  | 0.427 |  | 0.017 |  |
| Trial5 |  | Trial6 |  | 0.149 |  | 0.057 |  | 0.384 |  | 0.016 |  |
|  |  | Trial7 |  | 0.149 |  | 0.058 |  | 0.390 |  | 0.016 |  |
|  |  | Trial8 |  | 0.149 |  | 0.056 |  | 0.378 |  | 0.016 |  |
|  |  | Trial9 |  | 0.149 |  | 0.058 |  | 0.389 |  | 0.016 |  |
|  |  | Trial\_10 |  | 0.149 |  | 0.061 |  | 0.411 |  | 0.017 |  |
| Trial6 |  | Trial7 |  | 0.149 |  | 0.056 |  | 0.374 |  | 0.016 |  |
|  |  | Trial8 |  | 0.149 |  | 0.056 |  | 0.374 |  | 0.016 |  |
|  |  | Trial9 |  | 0.149 |  | 0.056 |  | 0.374 |  | 0.016 |  |
|  |  | Trial\_10 |  | 0.149 |  | 0.057 |  | 0.384 |  | 0.016 |  |
| Trial7 |  | Trial8 |  | 0.149 |  | 0.056 |  | 0.377 |  | 0.016 |  |
|  |  | Trial9 |  | 0.149 |  | 0.055 |  | 0.373 |  | 0.016 |  |
|  |  | Trial\_10 |  | 0.149 |  | 0.056 |  | 0.379 |  | 0.016 |  |
| Trial8 |  | Trial9 |  | 0.149 |  | 0.056 |  | 0.377 |  | 0.016 |  |
|  |  | Trial\_10 |  | 0.149 |  | 0.058 |  | 0.390 |  | 0.016 |  |
| Trial9 |  | Trial\_10 |  | 0.149 |  | 0.056 |  | 0.378 |  | 0.016 |  |
|  | | | | | | | | | | | |
|  |  |  |  |  |  |  |  |  |  |  |  |
| --- | --- | --- | --- | --- | --- | --- | --- | --- | --- | --- | --- |
| *Note.*  The posterior odds have been corrected for multiple testing by fixing to 0.5 the prior probability that the null hypothesis holds across all comparisons (Westfall, Johnson, & Utts, 1997). Individual comparisons are based on the default t-test with a Cauchy (0, r = 1/sqrt(2)) prior. The "U" in the Bayes factor denotes that it is uncorrected. | | | | | | | | | | | |
