# Supplementary material for: Cerebellum-mediated trainability of eye and head movements for dynamic gazing
Source: PLoS One. 2019 Nov 4;14(11):e0224458. doi: 10.1371/journal.pone.0224458 (PMC6827899; doi:10.1371/journal.pone.0224458)
Supplement: S8 File — (JASP) [file pone.0224458.s010.jasp › index.html]

JASP 


# Results

## Bayesian ANOVA

| Model Comparison - %change\_Ehratio | | | | | | | | | | | |
| --- | --- | --- | --- | --- | --- | --- | --- | --- | --- | --- | --- |
| Models | | P(M) | | P(M|data) | | BF M | | BF 10 | | error % | |
| Null model |  | 0.500 |  | 0.776 |  | 3.459 |  | 1.000 |  |  |  |
| Trial No |  | 0.500 |  | 0.224 |  | 0.289 |  | 0.289 |  | 4.633e -5 |  |
|  | | | | | | | | | | | |

### Post Hoc Tests

| Post Hoc Comparisons - Trial No | | | | | | | | | | | |
| --- | --- | --- | --- | --- | --- | --- | --- | --- | --- | --- | --- |
|  | |  | | Prior Odds | | Posterior Odds | | BF 10, U | | error % | |
| Trial1 |  | Trial2 |  | 0.149 |  | 0.076 |  | 0.509 |  | 1.653e -4 |  |
|  |  | Trial3 |  | 0.149 |  | 0.138 |  | 0.929 |  | 0.003 |  |
|  |  | Trial4 |  | 0.149 |  | 0.218 |  | 1.465 |  | 0.002 |  |
|  |  | Trial5 |  | 0.149 |  | 1.977 |  | 13.294 |  | 1.342e -4 |  |
|  |  | Trial6 |  | 0.149 |  | 19.857 |  | 133.538 |  | 6.834e -6 |  |
|  |  | Trial7 |  | 0.149 |  | 5.171 |  | 34.774 |  | 3.443e -5 |  |
|  |  | Trial8 |  | 0.149 |  | 0.269 |  | 1.806 |  | 0.002 |  |
|  |  | Trial9 |  | 0.149 |  | 0.179 |  | 1.203 |  | 3.102e -4 |  |
|  |  | Trial\_10 |  | 0.149 |  | 0.089 |  | 0.597 |  | 0.002 |  |
| Trial2 |  | Trial3 |  | 0.149 |  | 0.065 |  | 0.434 |  | 2.541e -4 |  |
|  |  | Trial4 |  | 0.149 |  | 0.071 |  | 0.475 |  | 7.335e -5 |  |
|  |  | Trial5 |  | 0.149 |  | 0.175 |  | 1.180 |  | 1.713e -4 |  |
|  |  | Trial6 |  | 0.149 |  | 0.566 |  | 3.803 |  | 1.554e -4 |  |
|  |  | Trial7 |  | 0.149 |  | 0.368 |  | 2.475 |  | 0.001 |  |
|  |  | Trial8 |  | 0.149 |  | 0.087 |  | 0.584 |  | 0.002 |  |
|  |  | Trial9 |  | 0.149 |  | 0.086 |  | 0.580 |  | 0.002 |  |
|  |  | Trial\_10 |  | 0.149 |  | 0.059 |  | 0.400 |  | 1.426e -4 |  |
| Trial3 |  | Trial4 |  | 0.149 |  | 0.054 |  | 0.364 |  | 6.079e -6 |  |
|  |  | Trial5 |  | 0.149 |  | 0.073 |  | 0.493 |  | 1.551e -5 |  |
|  |  | Trial6 |  | 0.149 |  | 0.136 |  | 0.914 |  | 0.003 |  |
|  |  | Trial7 |  | 0.149 |  | 0.114 |  | 0.768 |  | 0.005 |  |
|  |  | Trial8 |  | 0.149 |  | 0.057 |  | 0.386 |  | 6.596e -5 |  |
|  |  | Trial9 |  | 0.149 |  | 0.060 |  | 0.403 |  | 1.561e -4 |  |
|  |  | Trial\_10 |  | 0.149 |  | 0.054 |  | 0.363 |  | 6.618e -6 |  |
| Trial4 |  | Trial5 |  | 0.149 |  | 0.072 |  | 0.486 |  | 1.214e -5 |  |
|  |  | Trial6 |  | 0.149 |  | 0.141 |  | 0.950 |  | 0.002 |  |
|  |  | Trial7 |  | 0.149 |  | 0.116 |  | 0.778 |  | 0.005 |  |
|  |  | Trial8 |  | 0.149 |  | 0.056 |  | 0.380 |  | 3.794e -5 |  |
|  |  | Trial9 |  | 0.149 |  | 0.059 |  | 0.396 |  | 1.180e -4 |  |
|  |  | Trial\_10 |  | 0.149 |  | 0.054 |  | 0.365 |  | 4.366e -6 |  |
| Trial5 |  | Trial6 |  | 0.149 |  | 0.066 |  | 0.446 |  | 2.355e -4 |  |
|  |  | Trial7 |  | 0.149 |  | 0.062 |  | 0.417 |  | 2.246e -4 |  |
|  |  | Trial8 |  | 0.149 |  | 0.058 |  | 0.392 |  | 9.745e -5 |  |
|  |  | Trial9 |  | 0.149 |  | 0.055 |  | 0.367 |  | 4.718e -6 |  |
|  |  | Trial\_10 |  | 0.149 |  | 0.070 |  | 0.471 |  | 1.007e -4 |  |
| Trial6 |  | Trial7 |  | 0.149 |  | 0.054 |  | 0.364 |  | 6.157e -6 |  |
|  |  | Trial8 |  | 0.149 |  | 0.086 |  | 0.579 |  | 0.002 |  |
|  |  | Trial9 |  | 0.149 |  | 0.066 |  | 0.443 |  | 2.423e -4 |  |
|  |  | Trial\_10 |  | 0.149 |  | 0.112 |  | 0.755 |  | 0.005 |  |
| Trial7 |  | Trial8 |  | 0.149 |  | 0.078 |  | 0.522 |  | 3.667e -4 |  |
|  |  | Trial9 |  | 0.149 |  | 0.063 |  | 0.423 |  | 2.423e -4 |  |
|  |  | Trial\_10 |  | 0.149 |  | 0.100 |  | 0.671 |  | 0.005 |  |
| Trial8 |  | Trial9 |  | 0.149 |  | 0.055 |  | 0.367 |  | 4.689e -6 |  |
|  |  | Trial\_10 |  | 0.149 |  | 0.058 |  | 0.387 |  | 7.178e -5 |  |
| Trial9 |  | Trial\_10 |  | 0.149 |  | 0.060 |  | 0.403 |  | 1.587e -4 |  |
|  | | | | | | | | | | | |
|  |  |  |  |  |  |  |  |  |  |  |  |
| --- | --- | --- | --- | --- | --- | --- | --- | --- | --- | --- | --- |
| *Note.*  The posterior odds have been corrected for multiple testing by fixing to 0.5 the prior probability that the null hypothesis holds across all comparisons (Westfall, Johnson, & Utts, 1997). Individual comparisons are based on the default t-test with a Cauchy (0, r = 1/sqrt(2)) prior. The "U" in the Bayes factor denotes that it is uncorrected. | | | | | | | | | | | |
