# Supplementary material for: Cerebellum-mediated trainability of eye and head movements for dynamic gazing
Source: PLoS One. 2019 Nov 4;14(11):e0224458. doi: 10.1371/journal.pone.0224458 (PMC6827899; doi:10.1371/journal.pone.0224458)
Supplement: S9 File — (JASP) [file pone.0224458.s011.jasp › index.html]

JASP 


# Results

## Bayesian ANOVA

| Model Comparison - %change\_EHratio | | | | | | | | | | | |
| --- | --- | --- | --- | --- | --- | --- | --- | --- | --- | --- | --- |
| Models | | P(M) | | P(M|data) | | BF M | | BF 10 | | error % | |
| Null model |  | 0.500 |  | 0.986 |  | 72.199 |  | 1.000 |  |  |  |
| Trial No |  | 0.500 |  | 0.014 |  | 0.014 |  | 0.014 |  | 5.596e -5 |  |
|  | | | | | | | | | | | |

### Post Hoc Tests

| Post Hoc Comparisons - Trial No | | | | | | | | | | | |
| --- | --- | --- | --- | --- | --- | --- | --- | --- | --- | --- | --- |
|  | |  | | Prior Odds | | Posterior Odds | | BF 10, U | | error % | |
| Trial1 |  | Trial2 |  | 0.149 |  | 0.065 |  | 0.439 |  | 0.017 |  |
|  |  | Trial3 |  | 0.149 |  | 0.069 |  | 0.464 |  | 0.017 |  |
|  |  | Trial4 |  | 0.149 |  | 0.057 |  | 0.380 |  | 0.016 |  |
|  |  | Trial5 |  | 0.149 |  | 0.056 |  | 0.373 |  | 0.016 |  |
|  |  | Trial6 |  | 0.149 |  | 0.055 |  | 0.373 |  | 0.016 |  |
|  |  | Trial7 |  | 0.149 |  | 0.055 |  | 0.373 |  | 0.016 |  |
|  |  | Trial8 |  | 0.149 |  | 0.056 |  | 0.373 |  | 0.016 |  |
|  |  | Trial9 |  | 0.149 |  | 0.057 |  | 0.381 |  | 0.016 |  |
|  |  | Trial\_10 |  | 0.149 |  | 0.056 |  | 0.378 |  | 0.016 |  |
| Trial2 |  | Trial3 |  | 0.149 |  | 0.058 |  | 0.389 |  | 0.016 |  |
|  |  | Trial4 |  | 0.149 |  | 0.060 |  | 0.405 |  | 0.017 |  |
|  |  | Trial5 |  | 0.149 |  | 0.056 |  | 0.378 |  | 0.016 |  |
|  |  | Trial6 |  | 0.149 |  | 0.057 |  | 0.384 |  | 0.016 |  |
|  |  | Trial7 |  | 0.149 |  | 0.057 |  | 0.381 |  | 0.016 |  |
|  |  | Trial8 |  | 0.149 |  | 0.057 |  | 0.386 |  | 0.016 |  |
|  |  | Trial9 |  | 0.149 |  | 0.056 |  | 0.373 |  | 0.016 |  |
|  |  | Trial\_10 |  | 0.149 |  | 0.056 |  | 0.374 |  | 0.016 |  |
| Trial3 |  | Trial4 |  | 0.149 |  | 0.065 |  | 0.434 |  | 0.017 |  |
|  |  | Trial5 |  | 0.149 |  | 0.058 |  | 0.392 |  | 0.016 |  |
|  |  | Trial6 |  | 0.149 |  | 0.060 |  | 0.405 |  | 0.017 |  |
|  |  | Trial7 |  | 0.149 |  | 0.059 |  | 0.398 |  | 0.016 |  |
|  |  | Trial8 |  | 0.149 |  | 0.061 |  | 0.407 |  | 0.017 |  |
|  |  | Trial9 |  | 0.149 |  | 0.057 |  | 0.382 |  | 0.016 |  |
|  |  | Trial\_10 |  | 0.149 |  | 0.057 |  | 0.383 |  | 0.016 |  |
| Trial4 |  | Trial5 |  | 0.149 |  | 0.056 |  | 0.377 |  | 0.016 |  |
|  |  | Trial6 |  | 0.149 |  | 0.056 |  | 0.376 |  | 0.016 |  |
|  |  | Trial7 |  | 0.149 |  | 0.056 |  | 0.377 |  | 0.016 |  |
|  |  | Trial8 |  | 0.149 |  | 0.056 |  | 0.376 |  | 0.016 |  |
|  |  | Trial9 |  | 0.149 |  | 0.058 |  | 0.389 |  | 0.016 |  |
|  |  | Trial\_10 |  | 0.149 |  | 0.057 |  | 0.386 |  | 0.016 |  |
| Trial5 |  | Trial6 |  | 0.149 |  | 0.056 |  | 0.373 |  | 0.016 |  |
|  |  | Trial7 |  | 0.149 |  | 0.055 |  | 0.373 |  | 0.016 |  |
|  |  | Trial8 |  | 0.149 |  | 0.056 |  | 0.374 |  | 0.016 |  |
|  |  | Trial9 |  | 0.149 |  | 0.056 |  | 0.375 |  | 0.016 |  |
|  |  | Trial\_10 |  | 0.149 |  | 0.056 |  | 0.375 |  | 0.016 |  |
| Trial6 |  | Trial7 |  | 0.149 |  | 0.055 |  | 0.373 |  | 0.016 |  |
|  |  | Trial8 |  | 0.149 |  | 0.055 |  | 0.373 |  | 0.016 |  |
|  |  | Trial9 |  | 0.149 |  | 0.056 |  | 0.378 |  | 0.016 |  |
|  |  | Trial\_10 |  | 0.149 |  | 0.056 |  | 0.377 |  | 0.016 |  |
| Trial7 |  | Trial8 |  | 0.149 |  | 0.056 |  | 0.373 |  | 0.016 |  |
|  |  | Trial9 |  | 0.149 |  | 0.056 |  | 0.377 |  | 0.016 |  |
|  |  | Trial\_10 |  | 0.149 |  | 0.056 |  | 0.376 |  | 0.016 |  |
| Trial8 |  | Trial9 |  | 0.149 |  | 0.056 |  | 0.379 |  | 0.016 |  |
|  |  | Trial\_10 |  | 0.149 |  | 0.056 |  | 0.377 |  | 0.016 |  |
| Trial9 |  | Trial\_10 |  | 0.149 |  | 0.056 |  | 0.373 |  | 0.016 |  |
|  | | | | | | | | | | | |
|  |  |  |  |  |  |  |  |  |  |  |  |
| --- | --- | --- | --- | --- | --- | --- | --- | --- | --- | --- | --- |
| *Note.*  The posterior odds have been corrected for multiple testing by fixing to 0.5 the prior probability that the null hypothesis holds across all comparisons (Westfall, Johnson, & Utts, 1997). Individual comparisons are based on the default t-test with a Cauchy (0, r = 1/sqrt(2)) prior. The "U" in the Bayes factor denotes that it is uncorrected. | | | | | | | | | | | |
